# A high resolution A-to-I editing map in the mouse identifies editing events controlled by pre-mRNA splicing

**DOI:** 10.1101/gr.242636.118

**Published:** 2019-09

**Authors:** Konstantin Licht, Utkarsh Kapoor, Fabian Amman, Ernesto Picardi, David Martin, Prajakta Bajad, Michael F. Jantsch

**Affiliations:** 1 Center for Anatomy and Cell Biology, Medical University of Vienna, A-1090 Vienna, Austria;; 2 Institute of Theoretical Biochemistry, University of Vienna, A-1090 Vienna, Austria;; 3 Department of Biosciences, Biotechnologies, and Biopharmaceutics, University of Bari, I-70126 Bari, Italy;; 4 Institute of Biomembranes, Bioenergetics and Molecular Biotechnologies, National Research Council, I-70126 Bari, Italy

## Abstract

Pre-mRNA-splicing and adenosine to inosine (A-to-I) RNA-editing occur mostly cotranscriptionally. During A-to-I editing, a genomically encoded adenosine is deaminated to inosine by adenosine deaminases acting on RNA (ADARs). Editing-competent stems are frequently formed between exons and introns. Consistently, studies using reporter assays have shown that splicing efficiency can affect editing levels. Here, we use Nascent-seq and identify ∼90,000 novel A-to-I editing events in the mouse brain transcriptome. Most novel sites are located in intronic regions. Unlike previously assumed, we show that both ADAR (ADAR1) and ADARB1 (ADAR2) can edit repeat elements and regular transcripts to the same extent. We find that inhibition of splicing primarily increases editing levels at hundreds of sites, suggesting that reduced splicing efficiency extends the exposure of intronic and exonic sequences to ADAR enzymes. Lack of splicing factors NOVA1 or NOVA2 changes global editing levels, demonstrating that alternative splicing factors can modulate RNA editing. Finally, we show that intron retention rates correlate with editing levels across different brain tissues. We therefore demonstrate that splicing efficiency is a major factor controlling tissue-specific differences in editing levels.

Adenosine to inosine editing (A-to-I editing) deaminates adenosines in double-stranded RNAs leading to nucleotide differences between RNA and DNA (Licht and Jantsch 2016; Eisenberg and Levanon 2018). Inosine is primarily interpreted as guanosine by cellular machines (Basilio et al. 1962; Licht et al. 2019). Hence, adenosine deamination has a profound impact on the affected RNAs, changing their coding potential, base-pairing abilities, folding, mRNA-translation efficiency, or proteins associated with the targeted RNAs (Bass 2002; Tajaddod et al. 2015; Nishikura 2016; Licht et al. 2019).

A-to-I editing is mediated by adenosine deaminases acting on RNAs (ADARs). In mammals, two catalytically active ADAR enzymes, ADAR (ADAR1) and ADARB1 (ADAR2) are known. Both enzymes have overlapping, yet distinct substrate specificities (Bass 2002). Mice lacking *Adar* die at embryonic day 12.5, accompanied by liver disintegration, hematopoietic defects, and an increase in interferon signaling (Hartner et al. 2004, 2009; Wang et al. 2004). Lethality can be rescued when sensors of viral dsRNAs (*Ifih1* or *Mavs*) are also deleted (Mannion et al. 2014; Liddicoat et al. 2015; Pestal et al. 2015). Together, this suggests a role for *Adar* in innate immunity, possibly helping in discriminating self from nonself RNA (Mannion et al. 2014; Liddicoat et al. 2015; Pestal et al. 2015). *Adarb1* null mice die within 3 wk after birth but are rescued when a pre-edited *Gria2* allele is coexpressed (Brusa et al. 1995; Higuchi et al. 2000).

ADAR and ADARB1 require double-stranded RNA structures (dsRNA) for substrate recognition and editing (Higuchi et al. 1993; Herb et al. 1996). The sequence opposing the editing site is called the editing-complementary site (ECS). For exonic editing sites with an intronic ECS, editing needs to take place before mRNA-splicing (Higuchi et al. 1993; Herb et al. 1996). Consistently, Nascent-seq in *Drosophila* or human cells shows that the majority of editing takes place cotranscriptionally (Rodriguez et al. 2012; Hsiao et al. 2018). The efficiency of intron-removal has a direct impact on the level of editing and less efficient splicing generally leads to increased editing at sites that depend on an intronic ECS (Licht et al. 2016). Vice versa, editing can cause changes in alternative splicing: Intronic editing in the *Adarb1* transcript leads to the inclusion of a premature termination codon (Rueter et al. 1999). Moreover, splicing of the glutamate receptor is seemingly regulated by A-to-I editing (Higuchi et al. 2000; Schoft et al. 2007; Penn et al. 2013). Finally, loss of ADARs causes transcriptome-wide alternative splicing changes (Solomon et al. 2013; St Laurent et al. 2013; Mazloomian and Meyer 2015; Hsiao et al. 2018).

Editing levels increase during development and vary between tissues, a phenomenon that cannot be explained by differential expression of RNA editing enzymes alone (Wahlstedt et al. 2009; Stulić and Jantsch 2013; Huntley et al. 2016; Tan et al. 2017). For instance, editing of some but not all protein-coding sites is particularly high in human arteries (Tan et al. 2017). The possible underlying mechanisms are poorly understood but likely involve substrate-specific regulation but also global regulation at different levels (Marcucci et al. 2011; Garncarz et al. 2013; Tariq et al. 2013; Oakes et al. 2017; Tan et al. 2017).

A-to-I editing sites have been mostly identified in different human tissues (Levanon et al. 2004; Li et al. 2009b; Peng et al. 2012; Ramaswami et al. 2012, 2013; Bazak et al. 2014; Picardi et al. 2015). Most of the sites locate to noncoding regions of transcripts, especially UTRs and introns, and are deposited in various editing databases (Kiran et al. 2013; Ramaswami and Li 2014; Picardi et al. 2017). REDIportal, for instance, contains a collection of over 4.5 million human A-to-I editing sites (Picardi et al. 2017). In contrast, only ∼8000 editing sites have been deposited for mice (Ramaswami and Li 2014). Here, we used Nascent-seq to identify mouse editing sites. Moreover, by manipulating splicing, we unravel how A-to-I editing is regulated during transcript maturation and by mRNA-splicing.

## Results

### Nascent-seq identifies over 90,000 novel editing sites

Most mouse A-to-I editing sites known today locate to exonic regions. To explore the intronic editome, we used Nascent-seq (Menet et al. 2012; Rodriguez et al. 2012). To allow high fidelity editing-site identification, we aimed to compare wild-type and editing-null mice. To achieve this, *Adarb1^−/−^* mice were rescued by a pre-edited version of the glutamate receptor (*Adarb1^−/−^*, *Gria2^R/R^*) (Higuchi et al. 2000) and crossed with mice heterozygous for *Adar* (Hartner et al. 2004) carrying a homozygous *Mavs* deletion (*Adar^−/+^, Mavs^−/−^*). After crossing the heterozygous offspring, we selected for F2 mice of genotype *Adarb1^−/−^*, *Gria2*^R/R^, *Adar*^−/+^, *Mavs^−/−^* which are fully viable. Crossing of these mice to each other resulted in 25% mice carrying a homozygous deletion for *Adar*. These mice are smaller when compared to their heterozygous *Adar^−/+^* littermates and have a high mortality around day 15 after birth. For editing site determination, we isolated RNA at postnatal day 14 from editing-positive mice expressing ADAR and ADARB1: *Adar^+/+^, Mavs^−/−^, Adarb1^+/+^, Gria2^R/R^* (termed *wildtype* in this study). Secondly, we isolated RNA from editing-null mice (*double-knockout* or *dko* mice) where *Adar* and *Adarb1* have been deleted: *Adar^−/−^, Mavs^−/−^*, *Adarb1^−/−^*, *Gria2^R/R^*. Thirdly, we analyzed RNA from mice where only *Adarb1* (*Adar2*) had been deleted: *Adar^+/+^, Mavs^−/−^*, *Adarb1^−/−^*, *Gria2^R/R^* (called *Adarb1^−/−^* mice for simplicity).

**Figure 1.**
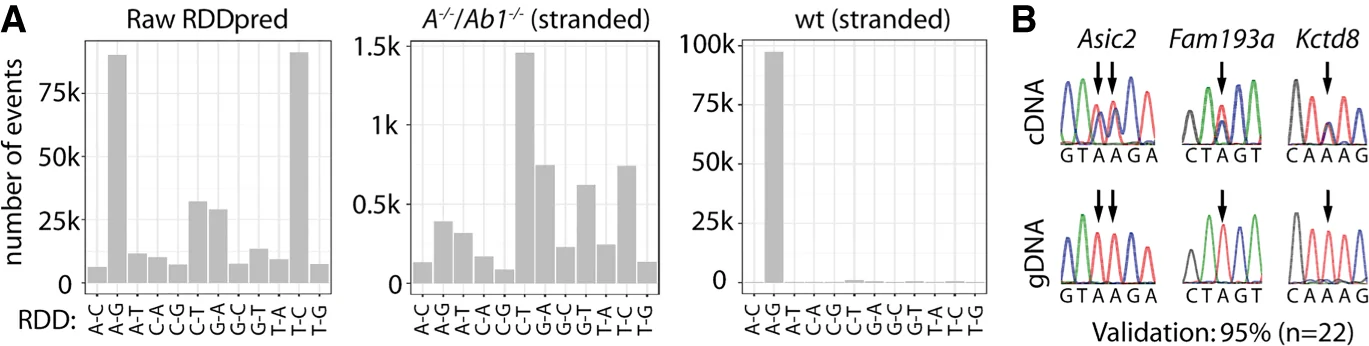
Nascent-seq identifies almost 100,000 mouse editing sites. (*A*) Nascent-RNA was prepared from brains of wild-type mice and subjected to Illumina sequencing (*n* = 3). After mapping to the mouse genome (mm10), potential RNA-DNA differences (RDDs) were determined using RDDpred (Raw RDDpred). To improve the identification of true RDDs, we removed all RDDs that either could not be mapped unambiguously to one strand, only occurred in one replica, or were also detected in an editing-deficient mouse line (*Adar^−/−^*, *Adarb1^−/−^*; *A^−/−^*, *Ab1^−/−^*). Thereby, we enriched for A-to-G RDDs indicative of A-to-I editing (wt stranded). (*B*) For validation, we used Sanger sequencing. Twenty-two editing sites were tested, and representative cDNA plus corresponding gDNA sequencing traces are shown (all traces are shown in Supplemental Fig. S2).

We extracted nascent RNA in triplicate from brains of wild-type and dko mice followed by depletion of polyadenylated transcripts and removal of rRNA to enrich for nascent transcripts. Nascent RNA was sequenced (125-bp, paired-end) yielding between 113 mio and 247 mio uniquely mapped reads per replicate and mapped to the mm10 RefSeq genome. Comparison with poly(A)-mRNA-seq shows an increased intronic coverage (Supplemental Fig. S1). Editing site detection was done using the RDDpred package (Kim et al. 2016). RNA-DNA differences (RDDs) were counted and plotted according to the type of difference resulting in the raw RDDpred output (Fig. 1A).

The majority of RDDs were A-to-G/T-to-C mismatches indicating A-to-I editing followed by C-to-T/G-to-A mismatches, suggesting C-to-U editing. All other transitions were found in relatively low numbers, demonstrating the reliability of the pipeline. Still, to further improve the quality, we removed all sites that were also detected in the double-knockout (*Adar^−/−^*, *Adarb1^−/−^*) or that could not be unambiguously aligned to one strand. This resulted in the wild type-only set (“wt/stranded”). Here, we almost exclusively observed A-to-G mismatches, suggesting enrichment for true A-to-I editing events. Using Sanger sequencing, we validated 21 out of 22 editing sites; i.e., 95% (Fig. 1B; Supplemental Fig. S2; Supplemental Tables S1, S2). As expected, we did not detect any A-to-G peak in the *Adar^−/−^*, *Adarb1^−/−^* set. This supports the notion that ADAR and ADARB1 are the only active editing enzymes. Subsequently, only A-to-G transitions exclusively detected in the wild type were considered for further analysis. Thereby, we identified almost 100,000 A-to-I editing sites, which we submitted to the REDIportal (http://srv00.recas.ba.infn.it/atlas/search_mm.html) (Picardi et al. 2017).

### Nascent editing sites primarily locate to introns and are associated with repeat elements

**Figure 2.**
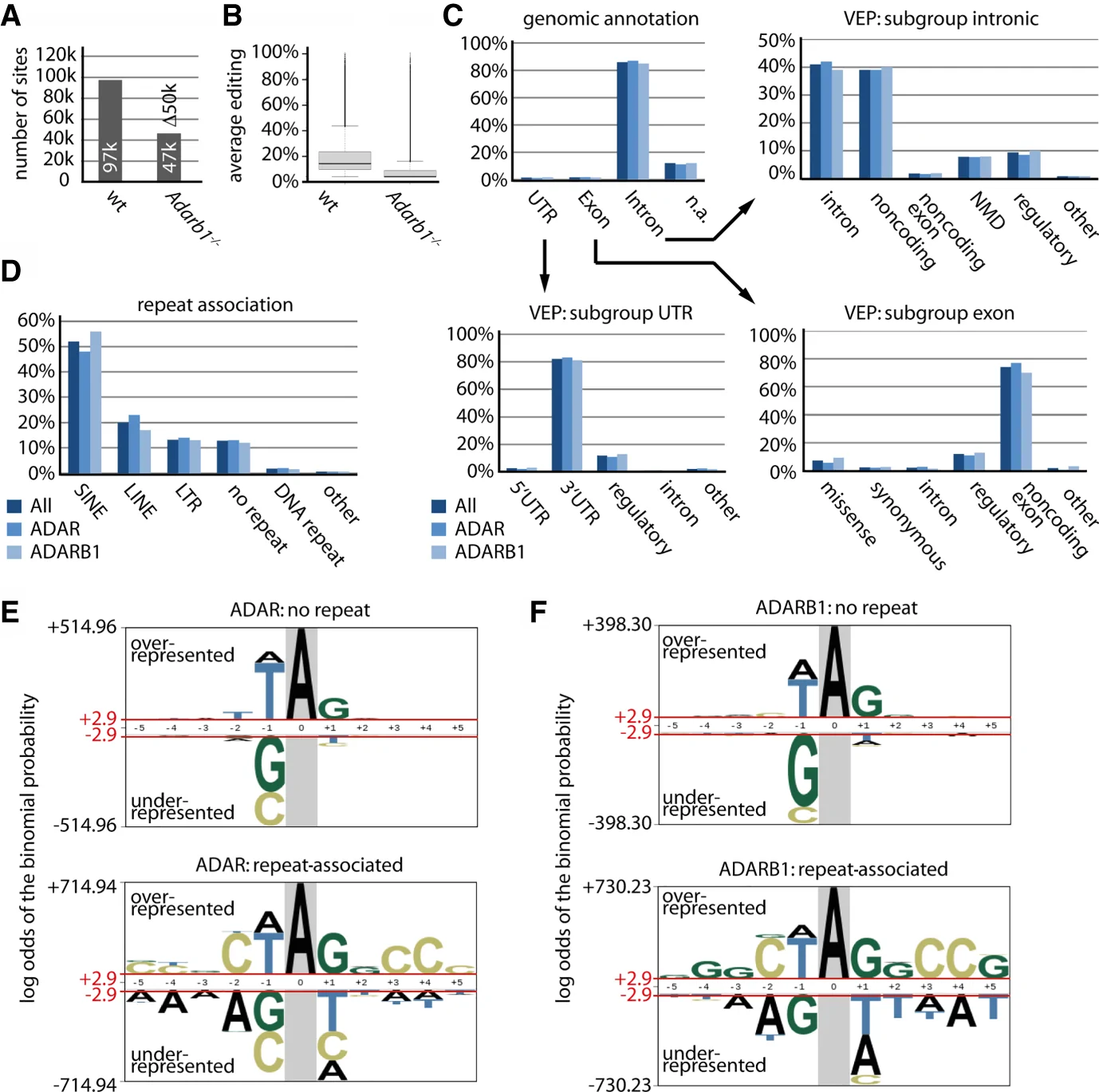
Both ADAR- and ADARB1-mediated editing is primarily associated with intronic regions and repeat elements. (*A*,*B*) *Adarb1* deletion leads to an ∼60% reduction in editing activity. (*A*) The total number of editing sites is shown separately for wild type and *Adarb1^−/−^* (sites edited by ADAR). (*B*) The average editing level of all identified editing sites is plotted for wild type and *Adarb1^−/−^* (overall ADAR editing activity). Error bars = standard deviation. *n* = 3. (*C*) The genomic annotation for all sites (dark blue), ADAR sites (blue), and ADARB1 sites (light blue) is given (Exon, Intron, UTR, [n.a.] not annotated/intergenic). In addition, for the subgroups Exon, Intron, and UTR, the predicted effect of editing is given using Ensembl's variant effect predictor (VEP). VEP terms: intron variant (intron); noncoding transcript variant (noncoding); noncoding transcript exon variant (noncoding exon); NMD transcript variant (NMD); regulatory region (regulatory); 5′ or 3′ UTR variant (5′ or 3′ UTR); missense variant (missense); synonymous variant (synonymous). (*D*) The percentage of editing sites associated with a particular repeat as identified by RepeatMasker is shown. Colors as in *C*. (*E*,*F*) Sequences enriched close to editing sites not associated with repeat elements (*upper* panel) or associated with repeat elements (*lower* panel) depicted separately for (*E*) ADAR or (*F*) ADARB1. The height of the nucleotide indicates either the degree of overrepresentation (*above* the line) or the degree of underrepresentation (*below* the line).

Compared to previous studies, we identified over 90,000 novel editing sites (Supplemental Fig. S3; Supplemental Table S3). Of 97,416 identified editing sites, approximately 50,000 are not edited in the *Adarb1^−/−^* mice, suggesting that they are primarily edited by ADARB1 (Fig. 2A), and ∼47,000 sites can be edited by ADAR. To estimate the overall ADAR and ADARB1 “editing activity”, we compared the editing level averaged across all sites in wild-type and *Adarb1^−/−^* mice (Fig. 2B).

The editing level in *Adarb1^−/−^* mice is more than threefold lower compared to wild type, suggesting that ADARB1 is the dominant editase in the mouse brain. Eighty-six percent of the editing sites map to intronic regions (Fig. 2C). The increased intronic coverage also fostered the identification of many lowly edited sites (Supplemental Fig. S4). Using the Ensembl variant effect predictor (VEP), we predicted the effect of editing events separately for exonic, intronic, and UTR sites (Fig. 2C; McLaren et al. 2016). The majority of exonic sites, for instance, are predicted to impact noncoding transcripts, followed by regulatory regions and missense (nonsynonymous) variants. Intronic editing sites frequently locate to noncoding transcripts and transcripts predicted to be NMD-targets. It should be noted that “noncoding transcript” also covers transcript variants that overlay with protein-coding genes. The majority of UTR editing occurs in 3′ UTRs. Some predictions appear to clash with the overlying genomic annotation (e.g., 2.2% of the exonic variants are predicted to have an effect on introns). This can be explained by multiple VEP annotations for one genomic site (see Methods). The proportions of genomic locations and effects predicted by VEP did not change when plotted separately for ADAR and ADARB1 sites. The majority of editing sites are associated with repeat elements, in particular, short interspersed nuclear elements (SINEs), long interspersed nuclear elements (LINEs), and long terminal repeats (LTRs) (Fig. 2D). Both ADAR and ADARB1 exhibit similar preferences toward different classes of repeat elements. Only SINEs are slightly enriched for ADARB1 editing, whereas LINEs are enriched for ADAR-mediated editing. Subsequently, we analyzed the nucleotides enriched or underrepresented around editing sites separately for ADAR and ADARB1 and split these into repeat-associated and nonrepeat-associated sites (Fig. 2E,F). The sequence-motif strongly differs with respect to repeat status but exhibits only small differences for ADAR and ADARB1 sites. This suggests that substrate preferences for both editases differ only slightly. However, based on the VEP prediction and repeat status, this only moderately impacts the overall preference for specific transcript features.

Editing-complementary sequences (ECSs) oppose editing sites and form a dsRNA with the editing region. To identify ECSs regions, we calculated the energetically most favorable hybridization site between the region ±15 nt around all editing sites identified in the Nascent-seq data and the extended surrounding region of ±2500 nts around the editing sites. Thereby, we predicted ∼50,000 ECSs (Supplemental Fig. S5A; Supplemental Table S3). To test the quality and significance of the predicted pairing, several assays were performed. First, we deduced an empirical *P*-value by comparing hybridization energy of the predicted ECSs to the energies calculated for 1000 input sequences with shuffled dinucleotides. An ECS was accepted at a *P*-value ≤0.001. As a control, we repeated the analysis with randomly selected adenosines having the same genomic features (e.g., “intronic” plus “repeat”). In contrast to true editing sites, randomly selected genomic regions did not allow identification of ECSs of similar quality (Supplemental Fig. S5B). Next, to independently confirm the predictions, we analyzed the sequence spanning the editing site to the predicted ECS using the RNA folding prediction tool RNAfold and the structure visualization tool *forna* (Lorenz et al. 2011; Kerpedjiev et al. 2015). The structures predicted for all 10 randomly selected transcripts again identified the proposed ECS (Supplemental Data Set S1). Lastly, for experimental validation, we cloned the genomic DNA coding for 10 editing sites with or without the corresponding predicted ECS into pcDNA3.1^−^ (Supplemental Data Set S1, Supplemental Table S4). Following cotransfection with a plasmid expressing FLAG-rADAR2, RT-PCR, and Sanger sequencing, we validated 10 out of 10 ECS predictions (Supplemental Fig. S6; Supplemental Table S5; Supplemental Methods).

**Figure 3.**
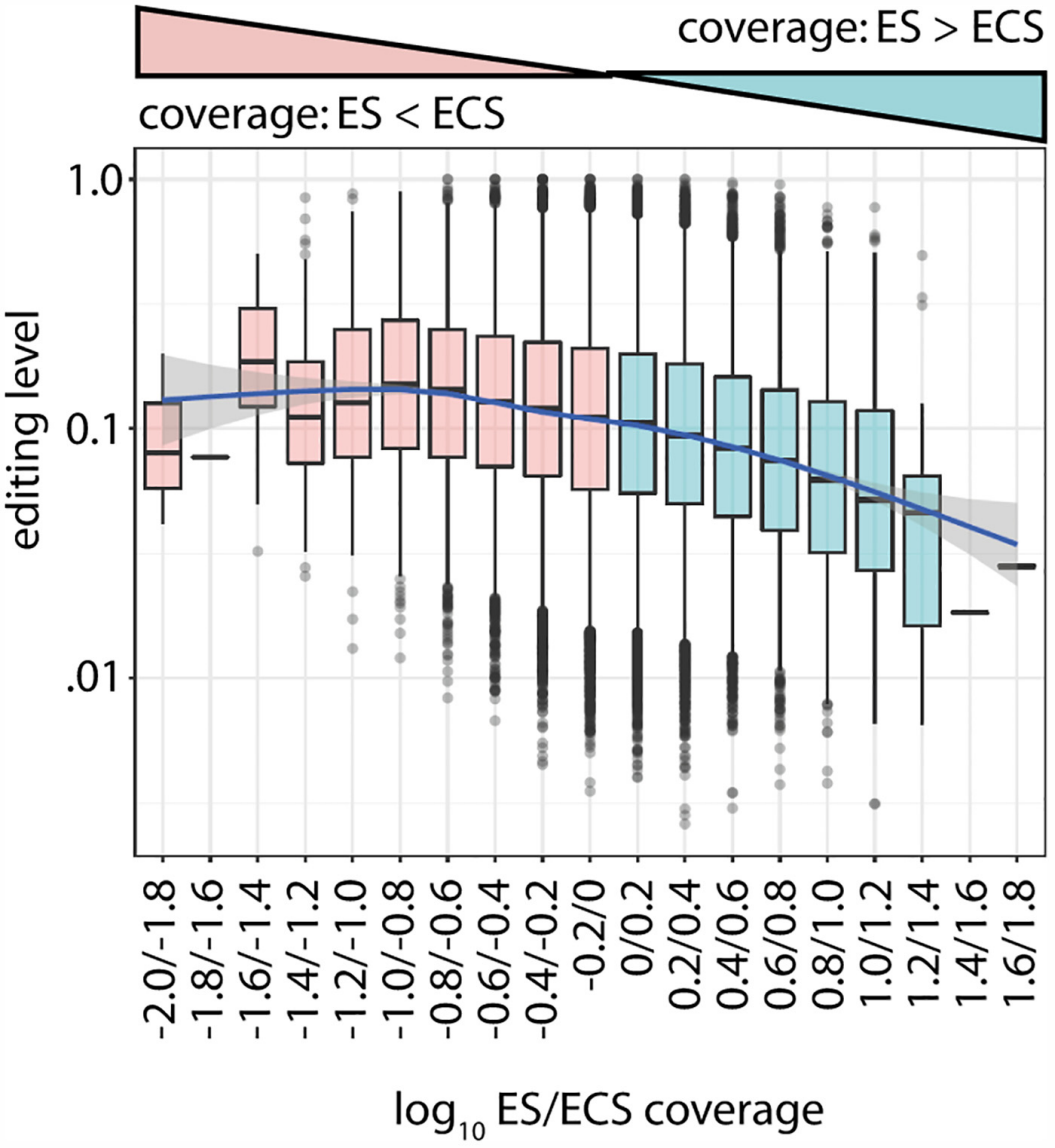
The persistence of the ECS increases editing levels. Box plot showing binned editing sites according to their log_10_ ES/ECS coverage (red: log_10_ ES/ECS coverage < 0 → ES saturated with ECS; blue: log_10_ ES/ECS coverage > 0 → ES deprived of ECS) and the respective editing level (*left* side).

The many intronic editing sites identified by Nascent-seq suggest that the majority of editing happens cotranscriptionally as seen before (Rodriguez et al. 2012; Hsiao et al. 2018). When we grouped editing sites according to the ratio of editing site coverage/ ECS coverage (number of reads mapping to the editing site/number of reads mapping to the ECS), we saw that editing sites with a lower ratio generally have a higher editing level and vice versa, suggesting that the co-occurrence of the ECS within the same transcript is a major determinant of editing levels. Such co-occurrence is likely modulated by transcription speed and processing efficiency (Fig. 3). This effect was particularly strong for editing sites within repeat elements (Supplemental Fig. S7).

### Editing levels are cotranscriptionally modified by the efficiency of splicing

By studying a selected set of exonic editing sites, we previously showed that splicing efficiency is a major factor controlling the level of editing (Licht et al. 2016). To determine the impact of splicing on editing on a transcriptome-wide scale, we used the splicing inhibitor meayamycin (Gao et al. 2013). Meayamycin inhibits SF3b, a part of spliceosomal U2 snRNP at low nM concentrations. Meayamycin was used on short term cultures of two primary cell types. Firstly, we used bone marrow of 6-wk-old mice and inhibited splicing using a concentration of 5 nM meayamycin for 6 h. Secondly, we treated primary neuronal cultures established from brains of mice at embryonic day e11.5 with 15 nM meayamycin for 6 h.

For isolation of poly(A)-selected RNA and cDNA library preparation, 5 + 5 or 6 + 6 (untreated plus treated) biological replicates of primary neurons and bone marrow, respectively, were used. Libraries were sequenced in a paired-end 125-bp mode, and reads were mapped to the mouse genome (mm10). Each replicate yielded between 63 mio and 279 mio uniquely mapped reads.

**Figure 4.**
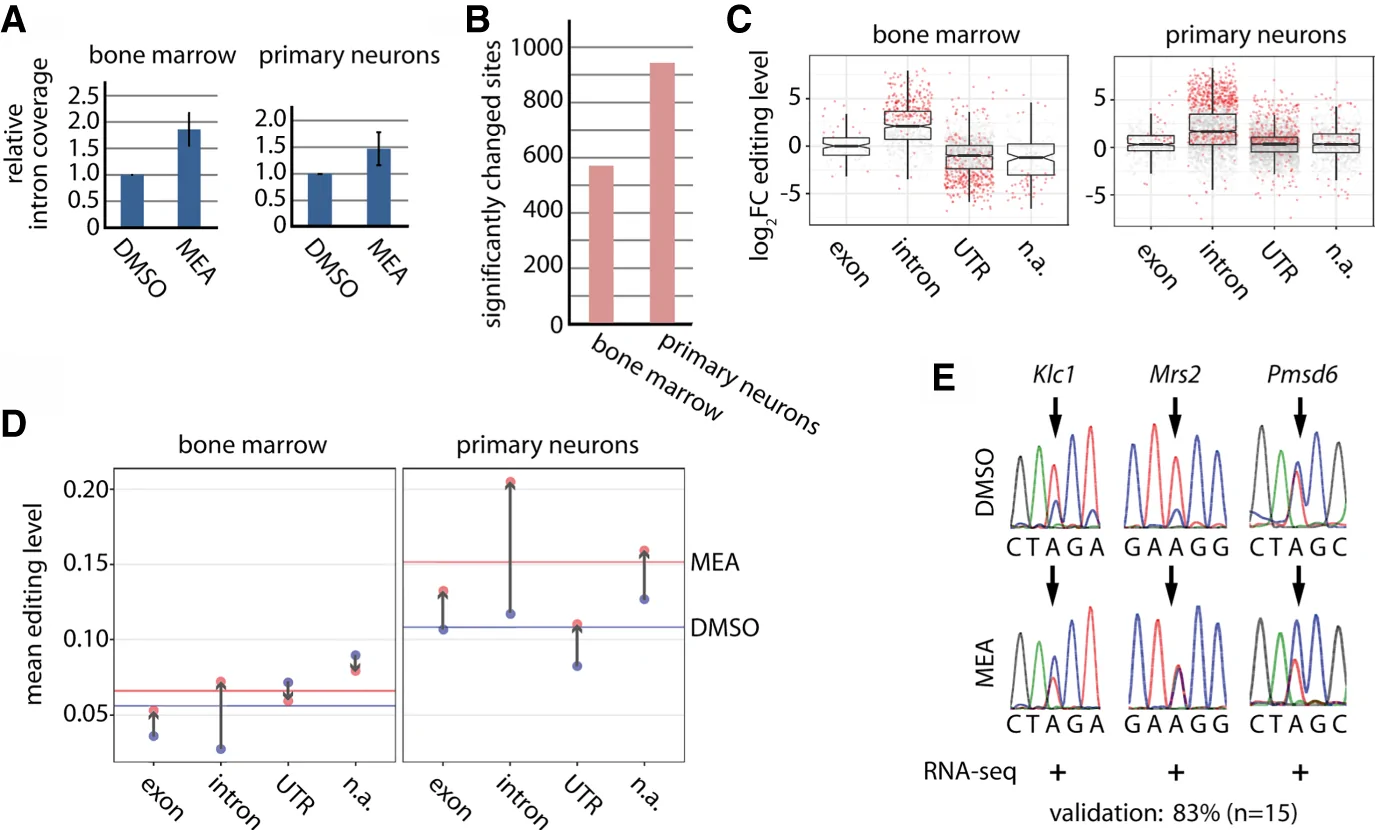
Reduced splicing efficiency globally increases exonic and intronic editing. (*A*) Either bone marrow cells or primary neurons were treated with the splicing inhibitor meayamcyin (MEA) or vehicle control (DMSO). RNA was isolated after treatment, and poly(A)-selected RNA was subjected to RNA-seq. The relative intronic coverage over editing sites after treatment with MEA is shown. Bone marrow: *n* = 6, primary neurons: *n* = 5, error bars = SEM. (*B*) Bar plot displaying the overall number of significantly changed editing sites for bone marrow and primary neurons. (*C*) Box plots showing the log_2_ fold change (log_2_FC) for editing levels in untreated (DMSO) and treated (MEA) primary cells (bone marrow: *left* panel, primary neurons: *right* panel) separated into different genic locations (exonic, intronic, UTR, [n.a.] not annotated, i.e., intergenic). Dots represent single editing sites. Significantly changed sites are highlighted in red (*P*-value < 0.05). (*D*) Mean editing levels in bone marrow (*left* panel) or primary neurons (*right* panel) for grouped editing sites (intron, exon, UTR, n.a.) under DMSO conditions (blue dots) or meayamycin treatment (red dots). The blue (DMSO) or red (meayamycin) line is drawn at the mean editing level of all sites. Gray arrows indicate the shift in mean editing levels upon meayamycin treatment. (*E*) Validation of changed editing levels by Sanger sequencing. The editing site is marked by an arrow. The change in editing determined by RNA-seq is given *below* the chromatograms (+ or − indicates an increase or decrease upon treatment as determined by RNA-seq).

Splicing inhibition works in both systems as evidenced by the larger proportion of reads mapping to intronic regions in the meayamycin-treated compared to control cells (Fig. 4A). Next, we quantified the number of reads at known editing sites (our Nascent-seq data plus known sites from the RADAR and DARNED databases [Kiran et al. 2013; Ramaswami and Li 2014]). We defined the editing level as the number of reads supporting editing divided by the total number of reads. We analyzed the bone marrow and the primary neuronal replicates (treated or untreated) separately and only considered editing sites that were supported by a minimum of five reads in each replicate of either the primary neuronal cultures or the bone marrow samples. Upon meayamycin treatment, 571 and 943 sites were found differentially edited (*P*-value < 0.05) in bone marrow and neuronal cultures, respectively (Fig. 4B; Supplemental Table S6). Editing generally increased upon treatment, most likely because splicing inhibition leads to a prolonged persistence of editing-competent dsRNA-structures formed within introns or between exons and introns (Fig. 4C). Consistently, the effect of splicing inhibition on editing is most pronounced for intronic and internal exonic sequences. In contrast, editing sites in UTRs are only modestly affected in the primary neuronal samples. Editing levels in bone marrow UTRs even decrease. A possible explanation could be that ADAR enzymes accumulate at intronic sequences and are depleted from UTRs under treatment conditions. This idea is supported by the analysis of the mean editing levels in meayamycin- and DMSO-treated cells (Fig. 4D). The basal editing levels (DMSO conditions) are lower in bone marrow (about 5%) than in primary neurons (about 10%). Under treatment conditions, the lower and thus potentially limiting editing activity in bone marrow may cause a shift of ADAR activity from UTR and not annotated sites to intronic and exonic sites. To further analyze this, we applied more stringent filtering using a cut-off of 10 reads minimum coverage and split editing sites into repeat-associated and nonrepeat-associated sites (Supplemental Fig. S8). Under these conditions, a similar shift in editing levels was observed, albeit the changes were stronger for repeat-associated sites. The position of the ECS played only a minor role, but the increase in editing was particularly strong for exonic sites in the bone marrow when the ECS was located in an intron, consistent with the notion that, in this scenario, the impact of splicing on editing levels should be particularly high (Supplemental Fig. S9). We validated the NGS results using Sanger sequencing with a validation rate of 83% (Fig. 4E; Supplemental Fig. S10). In sum, reduced splicing efficiency is associated with an increase in editing levels.

### Alternative splicing factors are a potential novel class of editing regulators

**Figure 5.**
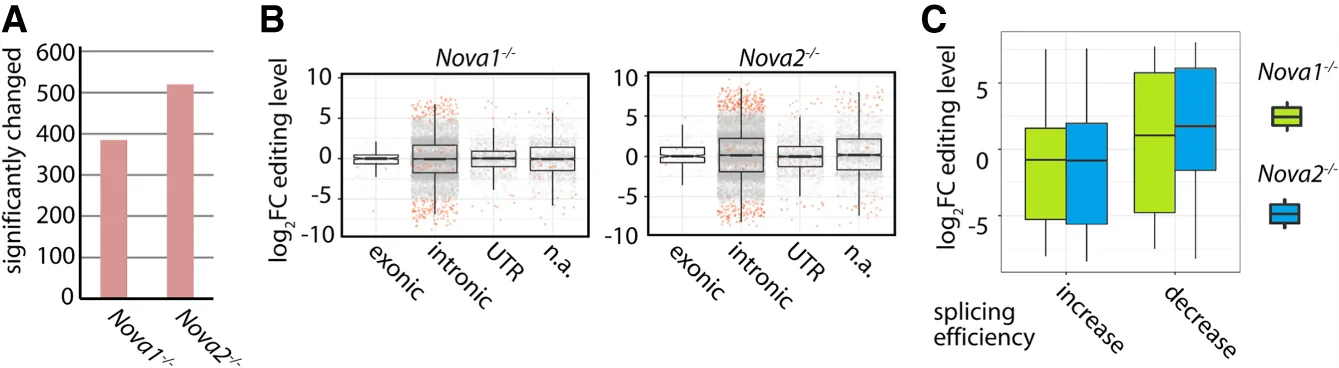
The alternative splicing factors NOVA1 and NOVA2 modulate editing levels. (*A*) Re-analysis of publicly available RNA-seq data from the cortices of six wild-type and three *Nova1^−/−^* and three *Nova2^−/−^* mice (Saito et al. 2016). The reads have been mapped to the mouse genome (mm10), and the editing level of known editing sites was determined. The total number of significantly changed editing sites is given (*P*-value < 0.05). (*B*) Editing levels in cortices of wild-type and either *Nova1^−/−^* (*left* panel) or *Nova2^−/−^* (*right* panel) knockout mice were determined, and the change in editing levels was plotted. Dots represent single editing sites. Significantly changed sites are highlighted in red (*P*-value < 0.05). A separate box plot is given for different genic locations (exonic, intronic, UTR, [n.a.] not annotated/intergenic). (*C*) Box plot showing the log_2_ fold change of editing levels for editing sites with up-regulated splicing efficiency (increase) or down-regulated splicing efficiency (decrease) for wild-type versus *Nova1^−/−^* (green) or wild-type versus *Nova2^−/−^* (blue) mice. Splicing efficiency is determined by a decrease (up-regulated splicing efficiency) or increase in intron-specific coverage (down-regulated splicing efficiency) as determined by *DEXSeq* (*P*-value < 0.1).

Next, we determined whether alternative splicing factors can affect editing levels. To this end, we analyzed RNA-seq data from cortices of *Nova1^−/−^* and *Nova2^−/−^* knockout mice (Saito et al. 2016). Both NOVA1 and NOVA2 are brain-specific alternative splicing factors (Ule et al. 2006). After mapping the reads to the mouse genome, we again called editing levels at all known sites, requiring a minimum of five reads in each replicate. Using these criteria, we detected 15,103 sites that were edited in any of the genotypes (Supplemental Table S7). In *Nova1*^−/−^ and *Nova2*^−/−^ mice, 385 and 520 editing sites, respectively, were differentially edited as compared to wild type (*P*-value < 0.05) (Fig. 5A). Depending on the location of the NOVA-binding motif in the pre-mRNA, NOVA proteins can act as silencers or enhancers of splicing (Ule et al. 2006). Consequently, the overall level of editing was not shifted, suggesting that NOVA proteins rather modulate editing in a target-specific way, unlike in meayamycin-treated cells (Fig. 5B). To test this idea further, we next analyzed if the change in editing levels for individual sites (where we had identified an ECS) corresponded to the change in splicing efficiency. As a measure of splicing efficiency we determined the intron-specific read-coverage (more coverage = less efficient splicing; less coverage = more efficient splicing). To achieve this, we used *DEXSeq* (Anders et al. 2012) and applied a *P*-value of 0.1 (Fig. 5C). As expected, when the splicing efficiency was reduced as compared to wild-type mice, editing levels increased (and vice versa). Still, this general trend cannot be observed for all editing sites, suggesting that NOVA1 and NOVA2 also indirectly influence editing levels. Moreover, consistent with a binary role of NOVA1 and NOVA2 in alternative splicing, we did not observe a general increase in the rate of intron retention in the *Nova1^−/−^ or Nova2^−/−^* knockout mice (Supplemental Fig. S11).

### Splicing controls tissue-specific editing levels

**Figure 6.**
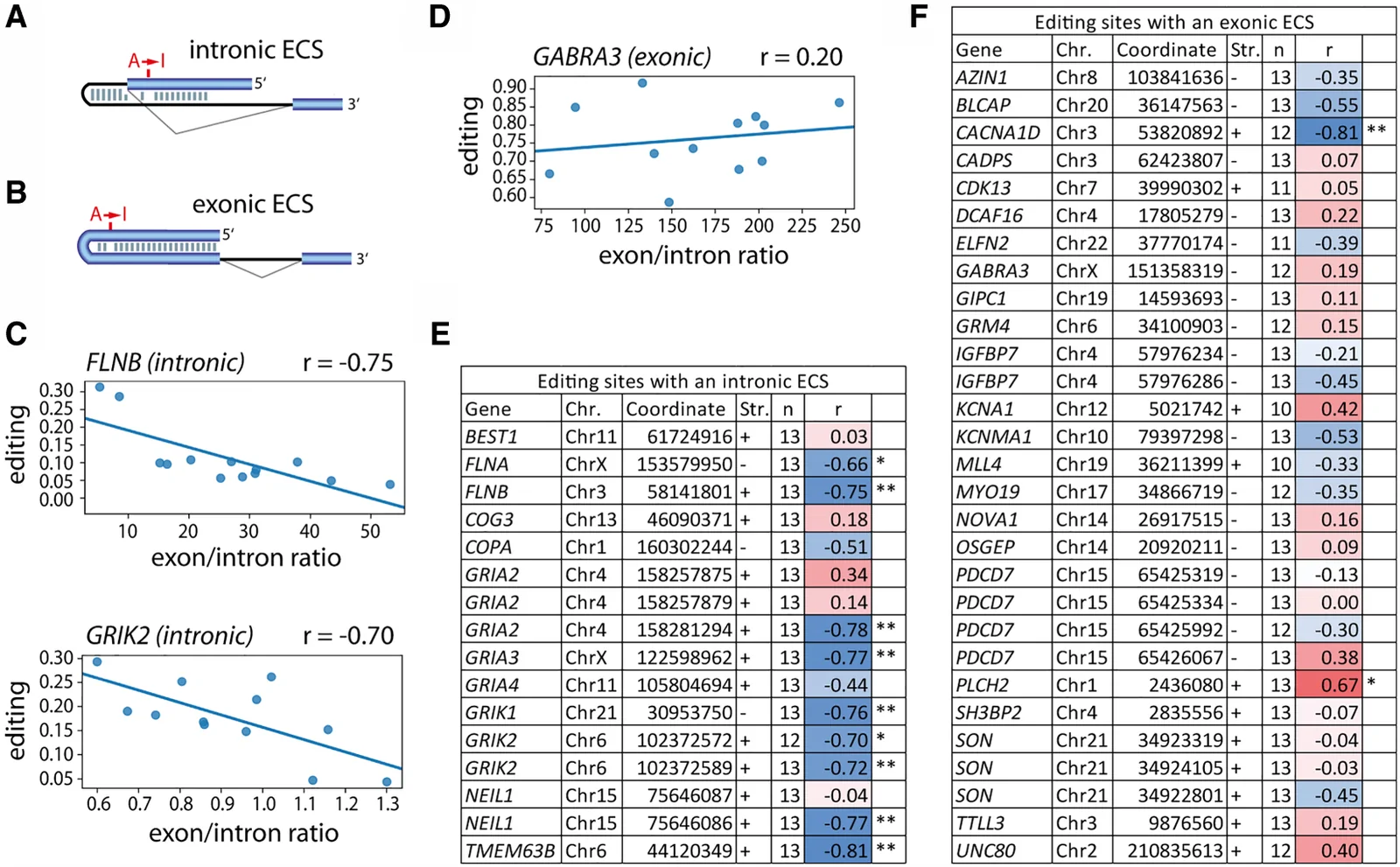
Splicing controls tissue-specific A-to-I editing for intron-dependent sites. (*A*,*B*) Exonic editing sites either depend on an (*A*) intronic editing complementary site (intronic ECS) or (*B*) exonic ECS. Exons are shown as blue bars, introns as thin lines. The editing site (A → I) is depicted. (*C*,*D*) Representative graphs for the correlation between average editing level and average exon/intron coverage at the downstream intron (as a measure of splicing efficiency) are shown for sites with a (*C*) intronic ECS like *FLNB* and *GRIK2* or (*D*) exonic ECS like *GABRA3* across different brain regions (RNA-seq data from GTEx). (*E*,*F*) The correlation (*r* = Pearson correlation coefficient) between average editing levels and average exon/intron ratio for conserved editing sites (Coordinates: hg19; [Chr.] chromosome; [Str.] strand) is plotted when the read coverage allowed calculation of editing levels and exon/intron ratio for at least 10 tissues (*n* = number of tissues) with minimum of two samples. Color code ranges from blue = strong negative correlation over white = no correlation to red = positive correlation. The significance is indicated by one or two asterisks: (*) *P*-value < 0.05, (**) *P*-value < 0.01.

Alternative splicing and intron retention levels vary between tissues and are important for defining tissue-specificity (Braunschweig et al. 2014; Baralle and Giudice 2017). Alternative splicing is enriched in the brain and regulated by brain-specific splicing factors including NOVA1 or NOVA2 (Raj and Blencowe 2015). We therefore reasoned that splicing efficiency may contribute to observed differences in editing between tissues. The impact of splicing on editing is particularly high when the editing site is coordinated with an intronic ECS (Licht et al. 2016). Most editing targets with an intronic ECS like *GRIA2* or *HTR2C* are primarily expressed in different brain regions (Li et al. 2009b; Heraud-Farlow et al. 2017). Therefore, we collected all RNA-seq data for 13 different brain regions available from the Genotype-Tissue Expression (GTEx) Consortium (The GTEx Consortium 2013). From these, we calculated the editing level for all exonic human editing sites available through the RADAR database that are conserved between human and either chimp, rhesus, or mouse (Ramaswami and Li 2014). As a measure of splicing efficiency, we calculated the level of intron retention by dividing the exonic by intronic read coverage. Next, we removed all editing sites where no editing was detected or no reads mapped to the intron. Moreover, we required both editing levels and splicing efficiency values to be calculated for at least 10 tissues supported by a minimum of two samples, leaving a total of 45 editing sites. Subsequently, editing sites were classified according to the location of the ECS as either intronic or exonic. We defined an ECS as intronic when the editing-competent duplex was formed between the edited exon and the downstream or upstream intron, whereas an exonic ECS contains an editing-competent dsRNA-stem solely within the edited exon (Fig. 6A,B). If the location of the ECS was not known, we identified the ECS using RNA secondary structure prediction tools (Lorenz et al. 2011; Kerpedjiev et al. 2015). Subsequently, we compared our structures to published predictions (if available) and found that all of them match (Supplemental Table S8). In sum, we classified 16 sites as intronic and 29 sites as exonic (Supplemental Table S8; Supplemental Data Set S2).

The expected negative correlation between editing levels and splicing efficiency was observed for editing sites with an intronic ECS like *FLNB* or *GRIK2* (Fig. 6C). In contrast, no correlation was observed at sites with an exonic ECS like *GABRA3* (Fig. 6D). Other editing sites with an intronic or exonic ECS exhibit a similar trend (Fig. 6E,F). This clearly indicates that editing levels in a particular tissue largely depend on splicing efficiency when the ECS is formed with the intron and identifies tissue-specific splicing as a major factor controlling editing.

## Discussion

In the mouse, only about 8000 A-to-I editing sites are known and cataloged (Ramaswami and Li 2014). Still, mice are an indispensable model system to study editing—for instance, to delineate the role of ADAR editing in innate immunity (Mannion et al. 2014; Liddicoat et al. 2015; Pestal et al. 2015). Using mice devoid in all active ADAR enzymes, we performed Nascent-seq to obtain a comprehensive set of nearly 100,000 A-to-I editing sites, most of which are novel. To our knowledge, this is the first study where a complete editing-null mouse has been used as a negative control to filter against false-positive editing sites.

ADARB2 (ADAR3) is a third member of the ADAR proteins (Chen et al. 2000). While the presence of a deaminase domain suggests that ADARB2 may have deaminating activity, no editing activity could be detected so far (Chen et al. 2000; Schneider et al. 2014). Still, it cannot be excluded that ADARB2 acts on unknown substrates. Here, we show on a transcriptome-wide scale that mice without both, ADAR and ADARB1, lack any significant mRNA-deamination activity, indicating that ADARB2 is not an active deaminase (see Fig. 1B). Transitions detected in the double-knockout mouse mostly reflect C-to-U editing but may also indicate RDDs that emerge due to unknown biological activity shortly after transcription (Wang et al. 2014).

Nascent-seq performed on brain tissue lacking only *Adarb1* showed an approximately threefold drop in editing in *Adarb1^−/−^* mice. This is consistent with previous findings showing higher expression of ADARB1 in the mouse brain as compared to ADAR (Heraud-Farlow et al. 2017). Apparently, ADAR can only partially compensate for the lack of ADARB1 editing activity. The analysis of ADAR- and ADARB1-mediated editing in the human GTEx data suggested that ADAR primarily edits repetitive sequences whereas ADARB1 is the primary enzyme targeting nonrepetitive coding sequences (Tan et al. 2017). In contrast, we show here that both ADAR and ADARB1 act on repeat elements. As we focus on the mouse brain, where ADARB1 is highly expressed, we cannot exclude that ADAR is primarily acting on repeats outside the nervous system. A potential drawback of our Nascent-seq approach is the lack of Nascent-seq data for the *Adar^−/−^* genotype. Therefore, we cannot clearly distinguish editing sites that could be edited by either ADAR or ADARB1 with equal efficiency. Such sites would be falsely identified as exclusive ADAR sites in our study. Also, if ADAR and ADARB1 needed to form heterodimers at particular sites, such sites would be falsely identified as exclusive ADARB1 sites. Thus, while we are confident that most sites are properly assigned as substrates of one of the two ADAR enzymes, our analysis clearly underestimates the number of sites where ADARB1 could act.

Using a mutational approach, we had shown previously that the efficiency of splicing is a key mechanism to control the level of editing at several selected sites (Licht et al. 2016). Here, using transcriptome-wide analysis, we can show that inhibition of splicing leads to a general increase of editing in intron-containing transcripts and mRNAs. The increase in editing upon splicing inhibition is particularly strong for exonic editing sites with adjacent intronic ECSs. Editing in UTRs does not follow a general trend, as a decrease was observed in bone marrow whereas UTR-editing in primary neurons was not shifted. Differences in basal editing activity between both primary cell systems may explain this. While the mean editing level in bone marrow is ∼5%, the level in primary neurons is almost twice as high. Moreover, ADARB1 is the primary editase in brain, while in bone marrow only ADAR is expressed at significant levels (Heraud-Farlow et al. 2017).

Consistent with the role of splicing on editing, altered editing patterns were detected in RNA-seq data from *Nova1^−/−^* or *Nova2^−/−^* knockout mice. NOVA1 and NOVA2 can either enhance splicing or silence splicing (Ule et al. 2006). Consistently, both increased and decreased editing levels were observed in *Nova1^−/−^* or *Nova2^−/−^* knockout mice. For most sites an increase in editing was accompanied with a drop in splicing efficiency and vice versa. Still, as this was not observed at all editing sites, NOVA1 and NOVA2 may also indirectly influence editing, for instance, by competing with other RNA-binding proteins. The splicing factor SRSF9 was found to repress ADARB1-mediated editing of brain-specific sites (Huang et al. 2018; Shanmugam et al. 2018; Quinones-Valdez et al. 2019). Moreover, a recent survey for the impact of RNA-binding proteins on editing levels revealed that alternative splicing factors have a great impact on editing levels (Quinones-Valdez et al. 2019). Together with our data, this suggests that alternative splicing factors are important editing regulatory factors that fine-tune editing levels similarly as established modulators of editing like AIMP2 and PIN1, or the E3 ligase WWP2 (Marcucci et al. 2011; Tan et al. 2017). Alteration of ADAR levels or its catalysis rate would obviously affect all substrates in one cell or tissue to the same extent. However, it is known that editing levels can vary in a substrate-specific manner between tissues. This is most clearly exemplified for the two RNAs encoding the paralogous proteins filamin A (FLNA) and filamin B (FLNB). While editing of the *Flna* transcript is highest in the cardiovascular system and the large intestine, editing of *Flnb* is low (Stulić and Jantsch 2013). Instead, *Flnb* editing levels are highest in the cartilaginous tissue and muscle (Czermak et al. 2018). Therefore, substrate-specific regulation must occur in these cases. RNA-binding proteins may compete with ADARs in a substrate-specific manner (Garncarz et al. 2013; Tariq et al. 2013; Quinones-Valdez et al. 2019). Our finding that splicing efficiency is a major factor in regulating editing levels may explain substrate-specific and tissue-specific regulation of editing levels. However, not all edited substrates follow the same trend. For instance, the editing level of the *Gria2* Q/R editing site does not correlate with reduced splicing efficiency. In contrast to other editing sites, editing of the *Gria2* Q/R site is close to 100% and required to support mammalian life (Higuchi et al. 2000). Seemingly, only edited *Gria2* pre-mRNA can be spliced efficiently to release the mature transcript (Higuchi et al. 2000; Schoft et al. 2007; Penn et al. 2013). This suggests a specific regulation of *Gria2* Q/R site editing and splicing that does not follow the general trend described here.

In sum, our study highlights the importance of cotranscriptional coordination between RNA editing and mRNA splicing and shows that splicing efficiency and splicing factors are major factors for the regulation of editing levels.

## Methods

### Isolation of nascent RNA and library preparation

*Mavs^+/−^* mice were acquired from Jackson Laboratory and crossed with *Adar^+/−^* and subsequently with *Adarb1^−/−^*, *Gria2*^R/R^ (stock #008634, allele: *Mavs*^*tm1Zjc*^). For Nascent-seq, mice of desired genotypes were sacrificed at p14, and nascent-RNA was isolated from brain tissue (Menet et al. 2012). After treatment with DNase I (Thermo Fisher Scientific), contaminating polyadenylated RNA was removed using the NEBNext poly(A) Isolation Module (New England Biolabs). Ribosomal RNA was removed using Ribo-Zero (Illumina). cDNA libraries were generated from 1 µg of nascent-RNA using NEBNext Ultra Directional RNA Library Prep kit for Illumina (New England Biolabs) and sequenced in paired-end mode with 125-bp read length on a HiSeq 2500 (Illumina).

### Splicing inhibition in bone marrow or primary neuronal cells

Bone marrow cultures were established as described (Kroeger et al. 2009; Licht et al. 2016). Bone marrow from one mouse was split and either treated with 5 nM meayamycin for 6 h or DMSO as vehicle control (Gao et al. 2013). Primary neuronal cultures were established from mouse embryos at e11.5. Each embryo isolate was split into two wells of a six-well dish coated with poly-D-lysine and cultured as established previously (Licht et al. 2016). One well was treated with 15 nM meayamycin for 6 h, while the corresponding well was treated with DMSO as control. RNA was isolated using TriFast (VWR Peqlab) and DNase I treated (Thermo Fisher Scientific). Libraries were generated as described above but from polyadenylated RNA isolated using the NEBNext poly(A) mRNA Magnetic Isolation Module (New England Biolabs).

### Nascent-seq data analysis

Sequenced reads were quality-trimmed and adapter-clipped using Trimmomatic (version 0.33) (Bolger et al. 2014) with default parameters. Quality was monitored using FastQC (version 0.11.3). Reads were mapped against the mouse reference genome (assembly mm10) using STAR (version 2.5.2b). RDDpred (Kim et al. 2016) was utilized for the detection of editing sites. MES-sites (Mapping Error-prone Sites; negative reference set) are provided for mm10 (http://epigenomics.snu.ac.kr/RDDpred/prior_data/Mouse.MES.txt.gz). Annotated editing sites from the databases DARNED (Kiran et al. 2013) and RADAR (Ramaswami and Li 2014) served as positive reference sites. DARNED and RADAR sites were converted to the mm10 coordinates using liftOver (Kuhn et al. 2013). Only RNA-DNA differences with a score ≥0.98 were considered. Subsequently, all sites overlapping with an annotated SNP, based on the variant annotation provided by the Mouse Genome Project (version 5) were removed (Keane et al. 2011). ES candidates exhibiting edited reads in at least two out of three WT samples, but in none of the dko samples, were classified as “WT only”. Conversely, sites with edited reads in at least two dko but none in WT samples were classified as “DKO only”. Editing sites were filtered and classified according to their strand topology. To this end, mapped paired-read files were split into separate half-samples containing only the first or second read in each pair. For each potential editing site, each half-sample was queried for observed substitution types considering the deriving strand. A substitution event was considered if it was observed in more than one read in more than two half-samples. We, and others before us (Levin et al. 2010), observed library-specific systematic error in the strand assignment of the reads (antisense shadow). Therefore, for sites with a relative antisense signal less than four times the global deduced antisense shadow, the signal from the less productive strand was ignored. To deduce the global antisense shadow, we applied the same strategy as described in Amman et al. (2018) for each chromosome and merged the single values by forming the average weighted by the chromosome length. The observed antisense shadows range between 0.34% and 1.74%. Integrating all this information, candidate editing sites were assigned a specific stranded substitution event or removed due to ambiguity (indicative of sequencing/mapping artifacts) or observation of corresponding substitutions on the opposing strand (indicative of a genomic variant). Finally, only unambiguous A → G events that occurred in the WT samples only were considered as true editing events. For all editing sites, the editing level was calculated using bam-readcounts (https://github.com/genome/bam-readcount). For follow-up analysis, editing sites supported by less than five reads were omitted. For ADARB1 knockout samples, editing sites were treated as edited if edited reads could be observed in more than one (out of three) samples or had a cumulative editing level greater than 1%. The obtained editing sites were characterized with respect to their genomic context (GENCODE basic annotation; release 17). Each ES was assigned to be either located in an UTR, exon, intron, or intergenic. If more than one classification applied to one ES, the higher ranked (UTR > exon > intron > intergenic) was selected. Subsequently, the potential impact of the observed A → G substitution was interrogated using Ensembl's variant predictor tool (VEP). If multiple effects for one substitution were predicted, only one variant was reported according to the following ranking: missense_variant > synonymous_variant > stop_lost > stop_ retained_variant > splice_acceptor_variant > splice_donor_variant > splice_region_variant > TF_binding_site_variant > regulatory_ region_variant > mature_miRNA_variant > 5_prime_UTR_variant > 3_prime_UTR_variant > non_coding_transcript_exon_variant > non_coding_transcript_variant > NMD_transcript_variant > intron_ variant > downstream_gene_variant > upstream_gene_variant > intergenic_variant. For a description of the variants see: www.ensembl.org/info/genome/variation/prediction/predicted_data.html#consequences.

To characterize the repeat status of an ES, RepeatMasker was used (Smit et al. 2013–2015) (www.repeatmasker.org; mm10; Repeat Library 20140131).

To calculate hybridization energies between an editing site and a potential editing complementary site (ECS), the program RNAplex was used (Tafer et al. 2011). To determine the read coverage of editing sites that are supported by at least five reads in all samples and their respective ECS, BEDTools multicov was used (Quinlan and Hall 2010).

To test for sequence motifs, all sites were separated according to their repeat association and their ADAR, ADARB1 preference. For each subset, a region ±5 nucleotides around the editing site was analyzed using pLogo generator (https://plogo.uconn.edu/) (O'Shea et al. 2013). To construct a sequence background that reflects the underlying base composition as closely, for each editing site a random 100-nt sequence stretch randomly drawn from within the surrounding ±200 bases around the editing site was used.

### Validation of editing events using Sanger sequencing

For validation of editing events, Sanger sequencing on cDNA libraries and corresponding genomic DNA from the same individual was used. Editing sites with at least 10% editing (Deffit et al. 2017) were validated, as low levels of editing are difficult to discern in Sanger sequencing chromatograms (Eggington et al. 2011). Similarly, for the validation of the splicing inhibition data, only editing sites with a minimum of 20% difference in editing were considered for validation. The validation was done on previously generated cDNA libraries. One-half microliter of the library (or genomic DNA) was amplified in a 25-µL reaction using OneTaq Quick-Load 2× Master Mix with Standard Buffer (New England Biolabs) using the following PCR protocol: Initial denaturation: 1 min at 94°C, followed by 35 cycles of 94°C (30 sec), 58°C (30 sec), 68°C (30 sec), and a final elongation for 5 min. PCR products were separated using gel electrophoresis and purified by gel-elution. Sanger sequencing was done using the eluted PCR products and the reverse primer (oligonucleotide sequences: Supplemental Table S1). Geneious v11 (Biomatters) was used to analyze Sanger chromatograms. The percentage of editing is defined as the height of the G peak divided by the sum of the A + G peaks (in the case of the reverse primer: the height of the T peak divided by the sum of the T + C peaks).

### Analysis of the splicing inhibition and *Nova1/2* knockout cortices

Mapping and editing level detection was done as described above. Editing sites which are not supported by at least five reads in all samples were omitted. Differentially edited sites were determined applying a *t*-test with the Welch approximation for the degrees of freedom on the log_10_ values of the observed levels. Sites with an editing level of zero were set to 0.001 before applying the log_10_. Unmapped reads for *Nova1* and *Nova2* knockout mice (cortex) and corresponding wild-type controls were downloaded from the Gene Expression Omnibus (GEO) repository (GSE69711). Reads were adapter-trimmed using Trimmomatic (Bolger et al. 2014) and subsequently mapped to the mouse genome (mm10) using Bowtie 2 (Langmead and Salzberg 2012) with sensitive parameters (-L 20, -N 1). Finally, the output was processed using SAMtools to generate sorted and indexed BAM files (Li et al. 2009a). The level of editing for previously identified editing sites (Nascent-seq) was determined by counting the percent of G-reads (+strand) or C-reads (−strand) using a custom script relying on SAMtools. Differential editing was calculated with the same parameters as described for the meayamycin treatment.

To determine the effect of NOVA1 or NOVA2 deletion on splicing efficiency for individual introns, *DEXSeq* (Anders et al. 2012) was used. The original *DEXSeq* workflow was designed to test for differential exon usage between different conditions. To include also intron retention events, we modified the first step of the analysis pipeline in which a nonoverlapping exon reference annotation is produced from the gene annotation with *DEXSeq*'s own dexseq_prepare_annotation.py Python script. We forced the script to include also intron regions by providing for each annotated transcript (i.e., exon chain) additionally a complementary intron chain. In this step, the parameter “–aggregate no” was set to prevent genes sharing exons from being merged into aggregated genes. Furthermore, transcript parts shorter than 10 bases were removed. From there, the standard *DEXSeq* workflow was used of counting reads and testing for differential transcript part usage (in the original pipeline called “exon usage”) between the WT and the respective mutant. Eventually, this information was linked with the information on differential editing levels between the two conditions for all editing sites that exhibit significant differential editing (*P*-value < 0.1). The distribution of the log_2_FC of editing levels was plotted separately for editing sites located in an up- or down-regulated transcript part, according to the *DEXSeq* analysis.

### GTEx data

GTEx data were downloaded from the NCBI database of Genotypes and Phenotypes (dbGaP; https://www.ncbi.nlm.nih.gov/gap; accession number phs000424.v6.p1). For each RNA editing site, genomic coordinates of the exon including the editing event as well as coordinates of the flanking intron were extracted by using RefSeq annotations (downloaded from UCSC) and custom scripts. Such coordinates were provided in input (in SAF format) to the featureCounts tool (Liao et al. 2014) in order to compute the number of reads supporting individual exons and introns in 329 GTEx RNA-seq experiments from 13 different brain locations. RNA-seq reads were downloaded from the dbGaP database and aligned onto the human reference genome (hg19 assembly) by means of STAR (Dobin et al. 2013). Exonic and intronic read counts were used to calculate an intron retention (IR) score that takes into account the exon to intron expression ratio. Finally, for each RNA editing site, IR scores and editing levels were correlated across brain locations. RNA editing levels for GTEx brain tissues were downloaded from REDIportal database (Picardi et al. 2017). Calculations were performed using custom Python scripts. Coordinates in the REDIportal are from the hg19 assembly. Consistently, we used hg19 for GTEx data analysis. Protein-coding genes used for our analysis are well annotated in this release. Consequently, the use of hg38 does not improve the analysis.

## Data access

RNA-seq data from this study have been submitted to the European Nucleotide Archive (ENA; https://www.ebi.ac.uk/ena) under accession number PRJEB27264. A-to-I editing sites identified using Nascent-seq have been submitted to REDIportal (http://srv00.recas.ba.infn.it/atlas/search_mm.html). Sanger sequencing chromatograms and Python scripts are available as Supplemental Material (Supplemental_Chromatograms.zip; Supplemental_Scripts.zip).

## Supplementary Material

Supplemental Material
