# Supplementary material for: A high resolution A-to-I editing map in the mouse identifies editing events controlled by pre-mRNA splicing
Source: Genome Res. 2019 Sep;29(9):1453–63. doi: 10.1101/gr.242636.118 (PMC6724681; doi:10.1101/gr.242636.118)
Supplement: Supplemental Material [file supp_29_9_1453__index.html]

A high resolution A-to-I editing map in the mouse identifies editing events controlled by pre-mRNA splicing — Supplemental Material 

# A high resolution A-to-I editing map in the mouse identifies editing events controlled by pre-mRNA splicing

## Supplemental Material

- Supplemental\_Material.pdf
- Supplemental\_Chromatograms.zip
- Supplemental\_Scripts.zip
- Supplemental\_Table\_S3.xlsx
- Supplemental\_Table\_S6.xlsx
- Supplemental\_Table\_S7.xlsx
