# Supplementary material for: A high resolution A-to-I editing map in the mouse identifies editing events controlled by pre-mRNA splicing
Source: Genome Res. 2019 Sep;29(9):1453–63. doi: 10.1101/gr.242636.118 (PMC6724681; doi:10.1101/gr.242636.118)
Supplement: Supplemental Material [file supp_gr.242636.118_Supplemental_Material.pdf]

Supplemental Material

A high resolution A-to-I editing map in the mouse identifies editing events controlled by pre-mRNA splicing

Konstantin Licht[1], Utkarsh Kapoor[1], Fabian Amman[2], Ernesto Picardi[3,4], David Martin[1], Prajakta Bajad[1], and Michael F. Jantsch[1]

1) Center for Anatomy and Cell Biology
Medical University of Vienna
Schwarzspanierstrasse 17
A-1090 Vienna, AUSTRIA

2) Institute of Theoretical Biochemistry
University of Vienna
Waehringerstrasse, 17
A-1090 Vienna, AUSTRIA

3) Department of Biosciences, Biotechnologies, and Biopharmaceutics
University of Bari
Via Orabona, 4
I-70126 Bari, ITALY

4) Institute of Biomembranes, Bioenergetics and Molecular Biotechnologies
National Research Council
Via Amendola, 165A
I-70126 Bari, ITALY

Table of Contents

Supplemental Figures:	page 3-12
Supplemental Tables:	page 13-17
Supplemental Material and Methods:	page 17
Supplemental Datasets:	page 18-41
Supplemental References:	page 42

Supplemental Figures

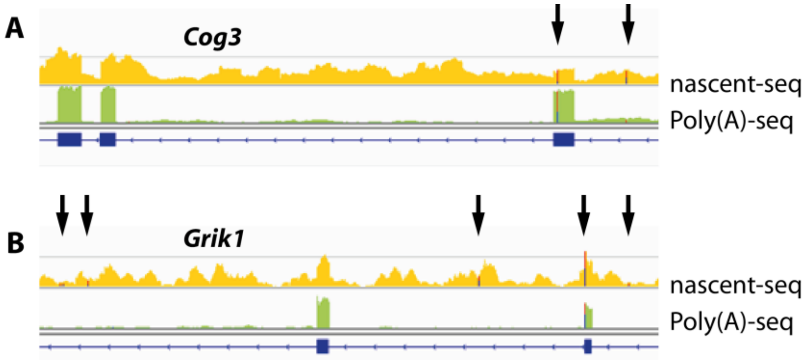

Supplemental Figure S1. The intronic coverage is higher for Nascent-seq than in poly(A) mRNA-seq. The coverage for two different edited transcripts (*A*) *Cog3*, and (*B*) *Grik1* is given for nascent-seq (yellow) and poly(A) mRNA-seq (green). The intron-exon structure of the gene is depicted below (blue); exons: bars; introns: thin lines. Editing sites are marked by arrows but can also be seen in the coverage profile (blue/red lines indicate mismatches).

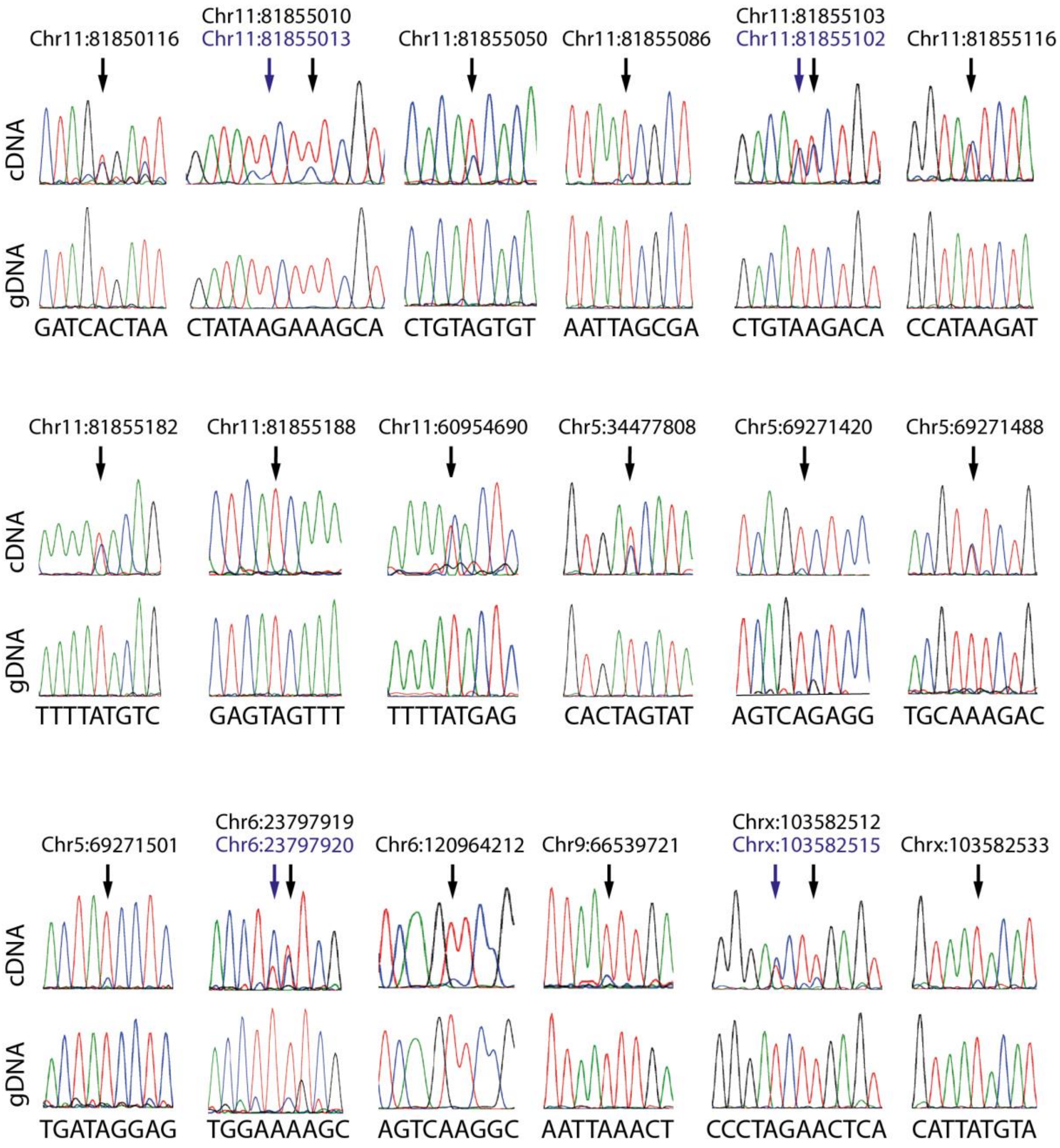

Supplemental Figure S2. Sanger sequencing of cDNA (top rows) and corresponding gDNA (bottom rows) for all 22 editing sites tested to validate the pipeline to identify editing sites. Editing sites are marked by an arrow. Coordinates of the editing sites are given above the arrow (mm10).

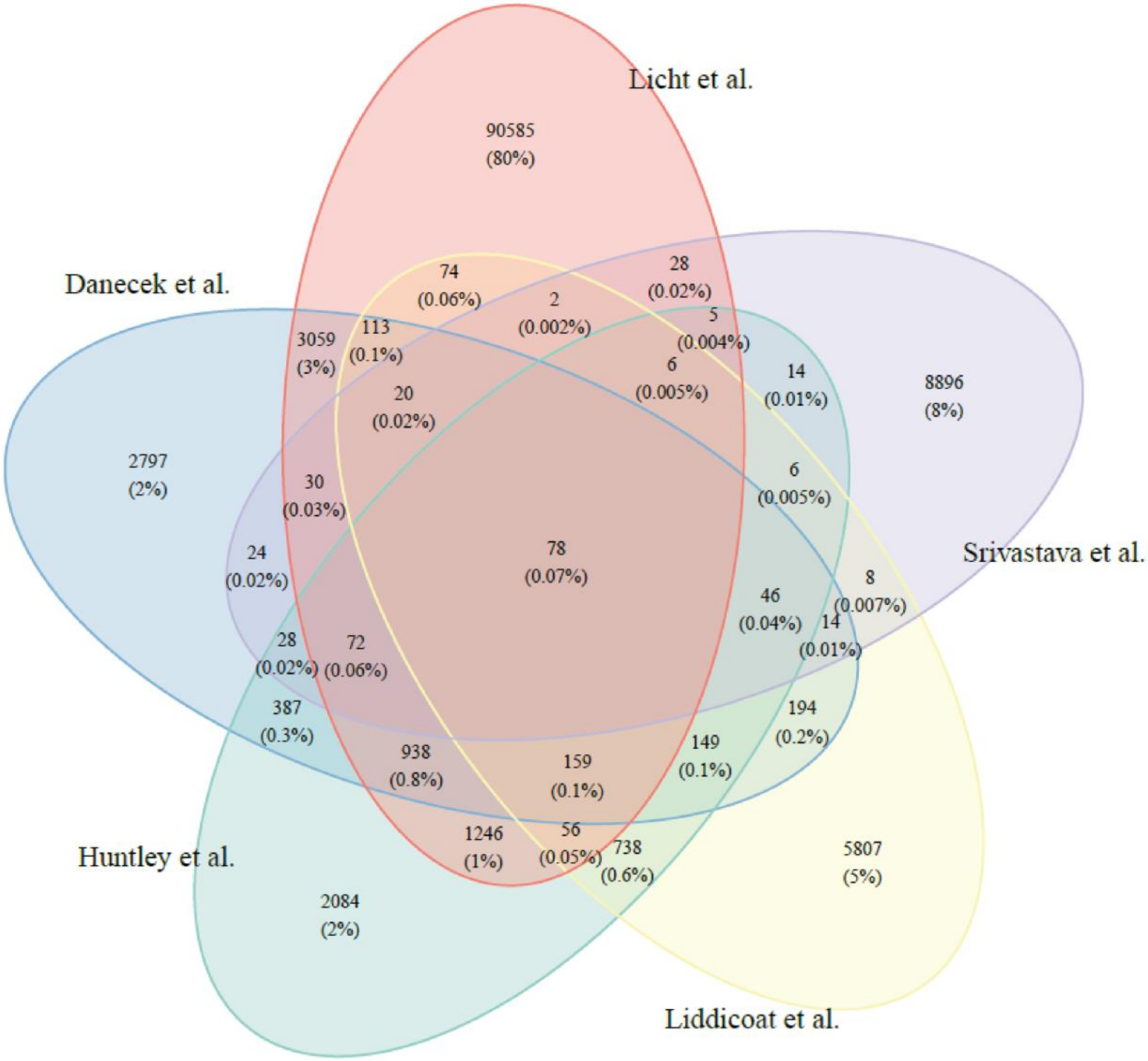

Supplemental Figure S3. Venn diagram showing the overlap of identified editing sites between this study and previous studies (Danecek et al. 2012; Huntley et al. 2016; Liddicoat et al. 2016; Srivastava et al. 2017).

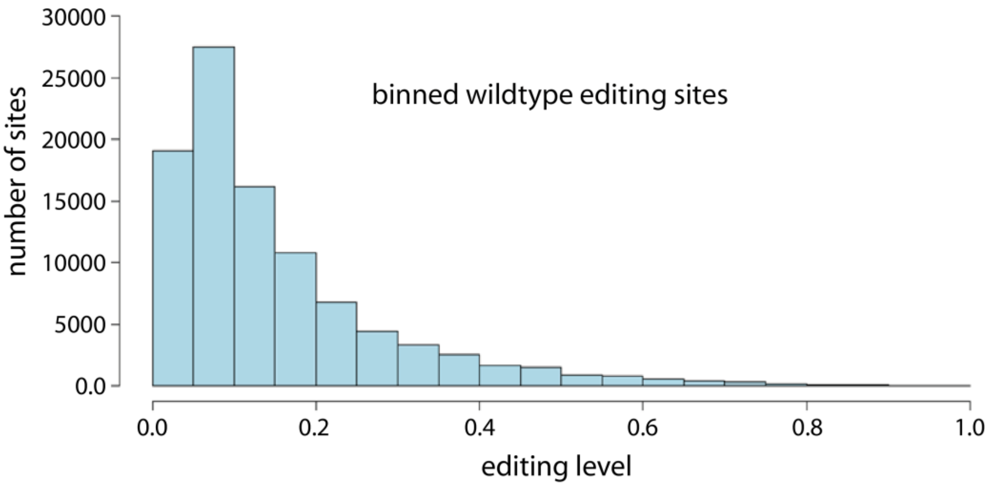

Supplemental Figure S4. The majority of editing sites identified using Nascent-seq in wildtype mice is edited below 20%. All editing sites have been binned according to their level of editing.

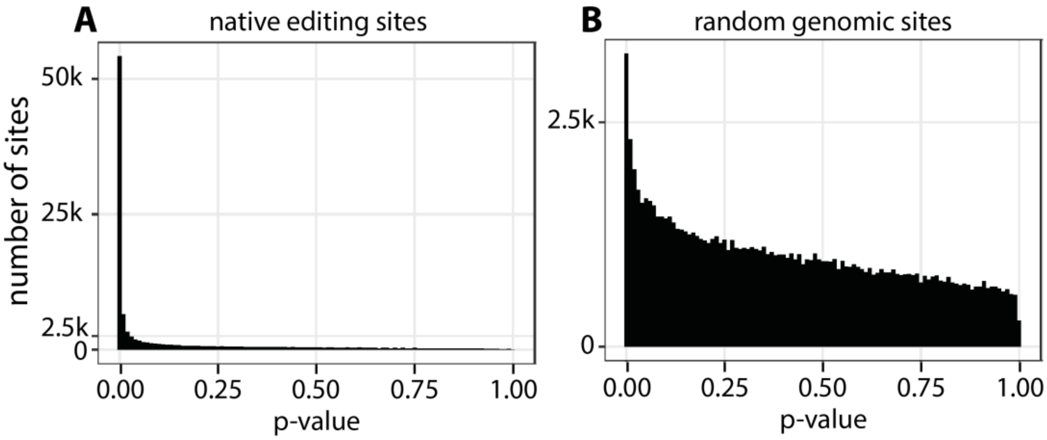

Supplemental Figure S5. (*A, B*) Comparison between bioinformatically predicted editing-complementary sequences (ECS) and randomized control sequences. (*A*) An empirical p-value was calculated by comparing the hybridization energy of the predicted ECS to the energies calculated from 1,000 dinucleotide shuffled input sequences. ECSs were accepted at a p-value level<=0.001. (*B*) To control the prediction, the same analysis was repeated with randomized editing site positions. Thereby, for each editing site an adenosine from the genome was randomly selected, but preserving its original genomic annotation and repeat status.

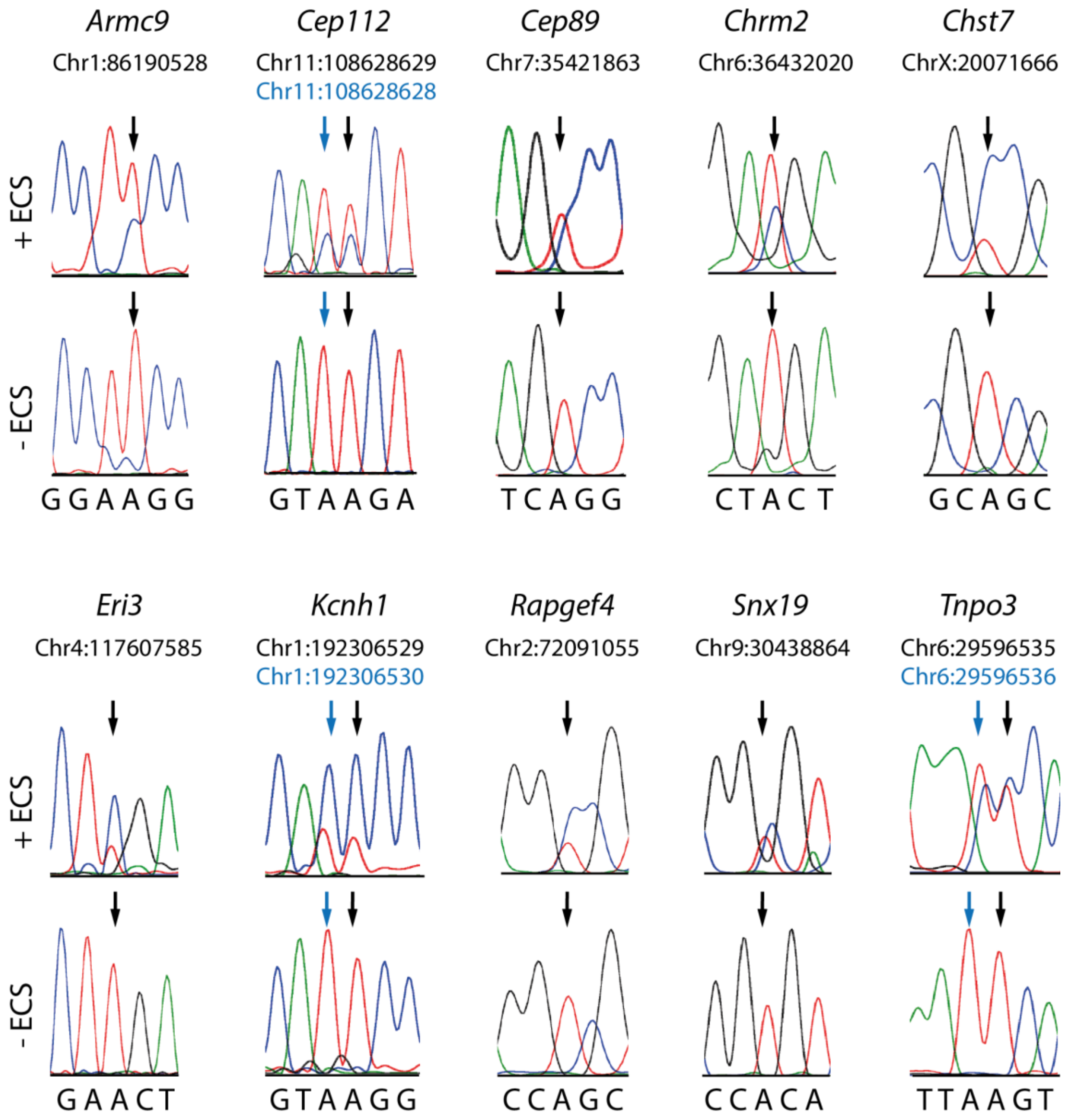

Supplemental Figure S6. Validation of bioinformatically predicted editing-complementary sequences (ECS). The genomic DNA encoding for edited transcripts and the corresponding predicted editing complementary sequence was cloned (+ ECS). Subsequently, the editing complementary region has been removed (the predicted secondary structure and the removed ECS (- ECS) regions is displayed in Supplemental Dataset S1). Following co-transfection with a plasmid coding for Flag-rADAR2 into HEK293 cells, RNA was isolated, reverse transcribed, amplified with target-specific primers, and submitted to Sanger sequencing. Genome coordinates: mm10.

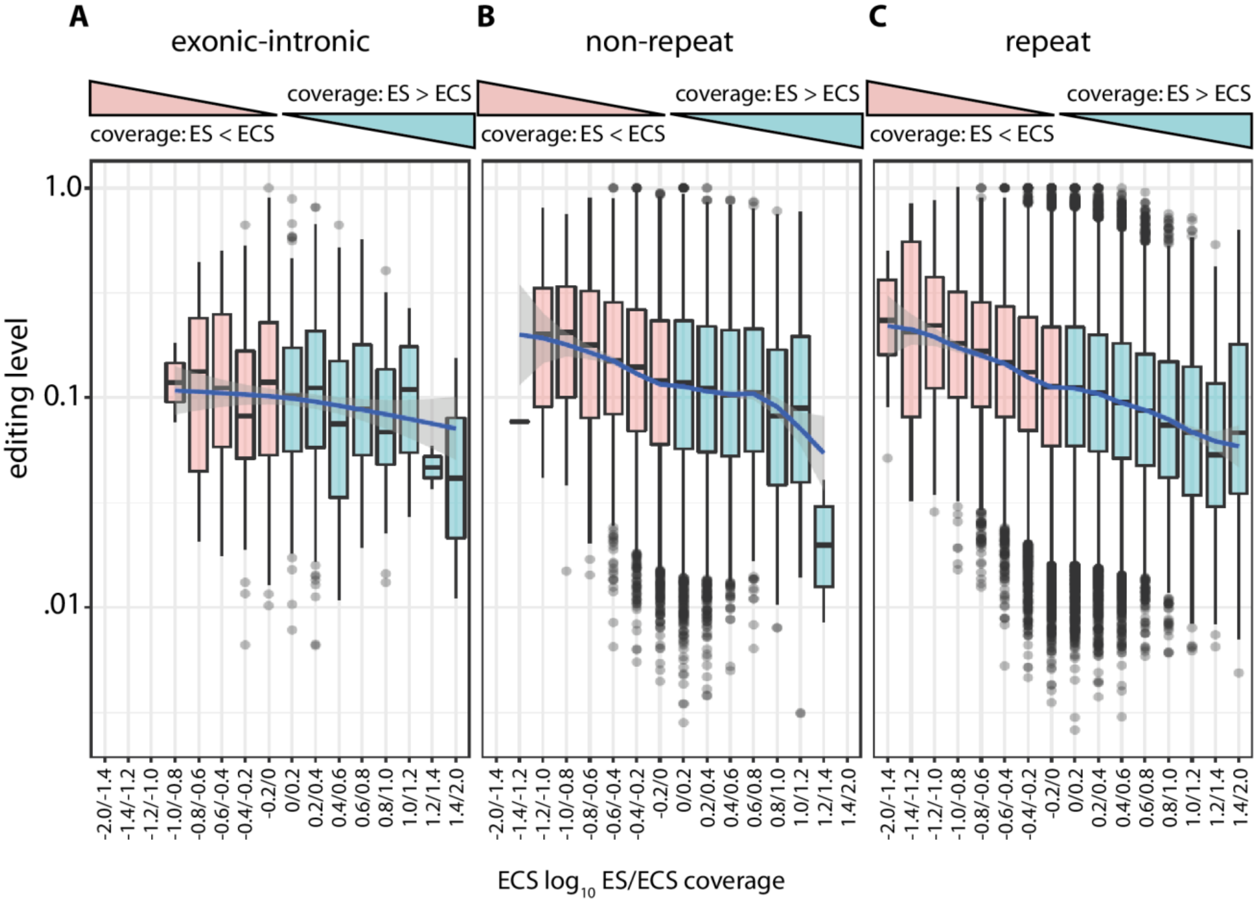

Supplemental Figure S7. The persistence of the ECS increases editing levels. *(A-C)* Boxplots showing binned editing sites according to their $\log_{10}$ ES/ECS coverage (red: $\log_{10}$ ES/ECS coverage<0 → ES saturated with ECS; blue: $\log_{10}$ ES/ECS coverage>0 → ES deprived of ECS) and the respective editing level (left side). The analysis was done separately for *(A)* exonic editing sites with an intronic ECS (exonic-intronic), for *(B)* non-repeat associated sites, and for *(C)* repeat associated sites.

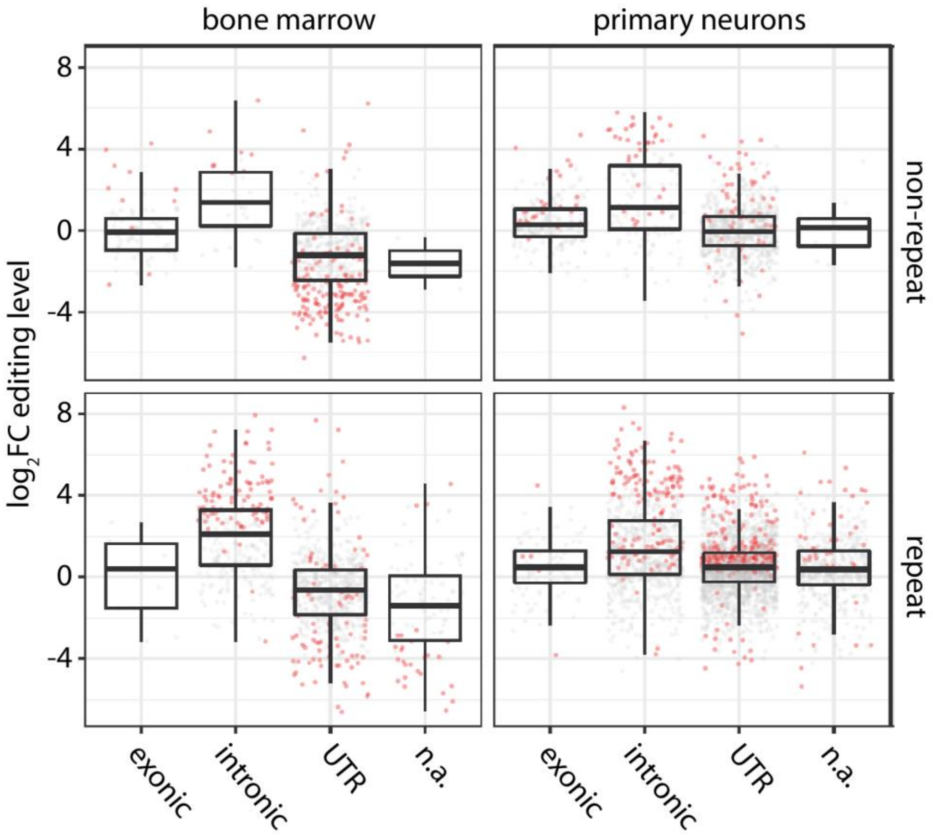

Supplemental Figure S8. Editing sites split according to their repeat status (compare to main figure 4). The editing sites were split according to their repeat status (upper panels: non-repeat, lower panels: repeat). Only sites with a minimum read coverage of 10 are shown.

Boxplots showing the log_2 fold change (log_2FC) for editing levels in untreated (DMSO) and treated (MEA) primary cells (left panels: bone marrow, right panels: primary neurons) separated into different genic locations as indicated below the panels (exonic, intronic, UTR, n.a.: not annotated/intergenic). Dots represent editing levels for individual sites that are changed significantly (red; p-value<0.05) or not significantly (grey; p-value>=0.05).

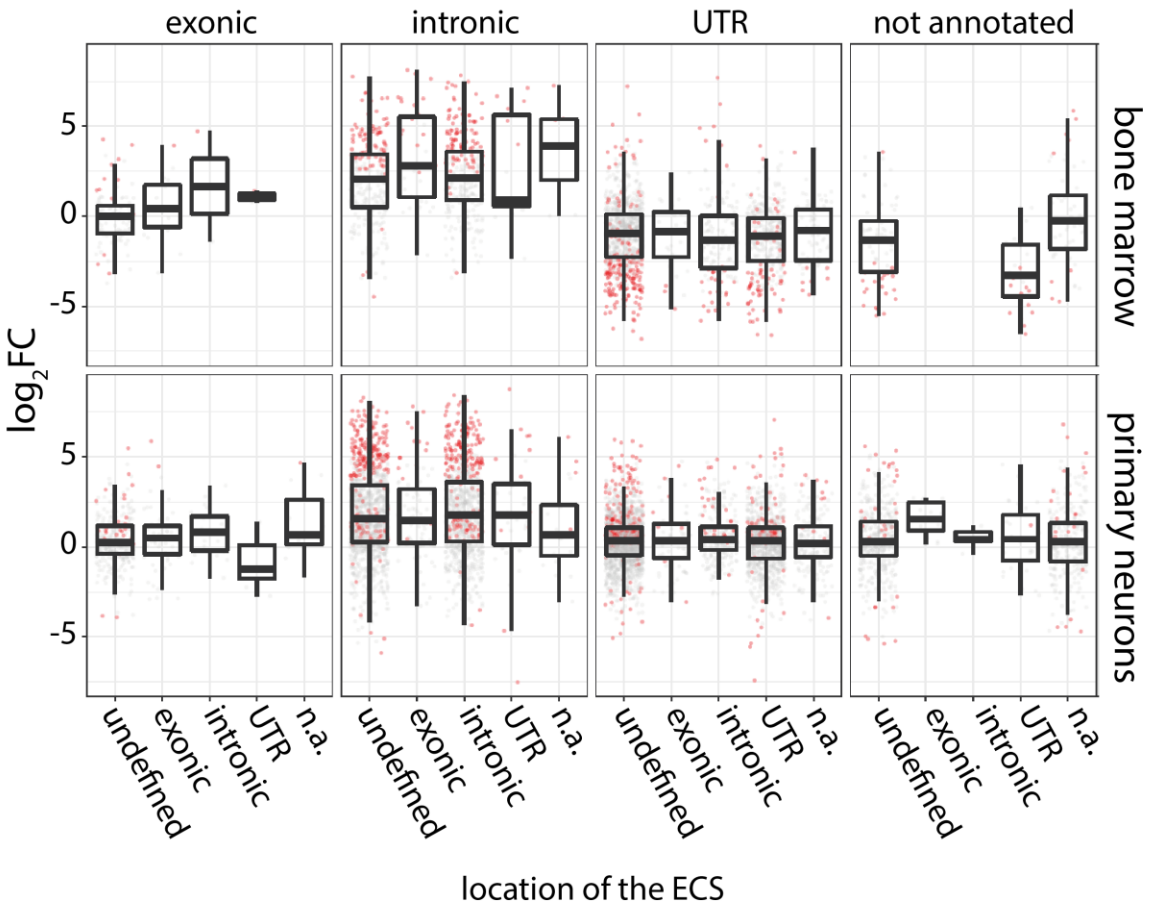

Supplemental Figure S9. Meayamycin treatment leads to increased editing levels in bone marrow and neuronal cells. Two primary cell types were treated with the splicing inhibitor meayamycin (MEA) or vehicle control (DMSO). RNA was isolated after treatment and poly(A)-selected RNA was subjected to RNA-seq. Boxplots showing the $\log_2$ fold change ($\log_2$FC) for editing levels in untreated (DMSO) and treated (MEA) primary cells (top panel: bone marrow, lower panel: primary neurons) separated into different genic locations as indicated on top of the panel (exonic, intronic, UTR, not-annotated i.e. intergenic). The location of the editing complementary site (ECS) is given below the panel (undefined: could not be identified; n.a.: not annotated/intergenic). Dots represent editing levels for individual sites that are changed significantly (red; p-value<0.05) or not significantly (grey; p-value>=0.05).

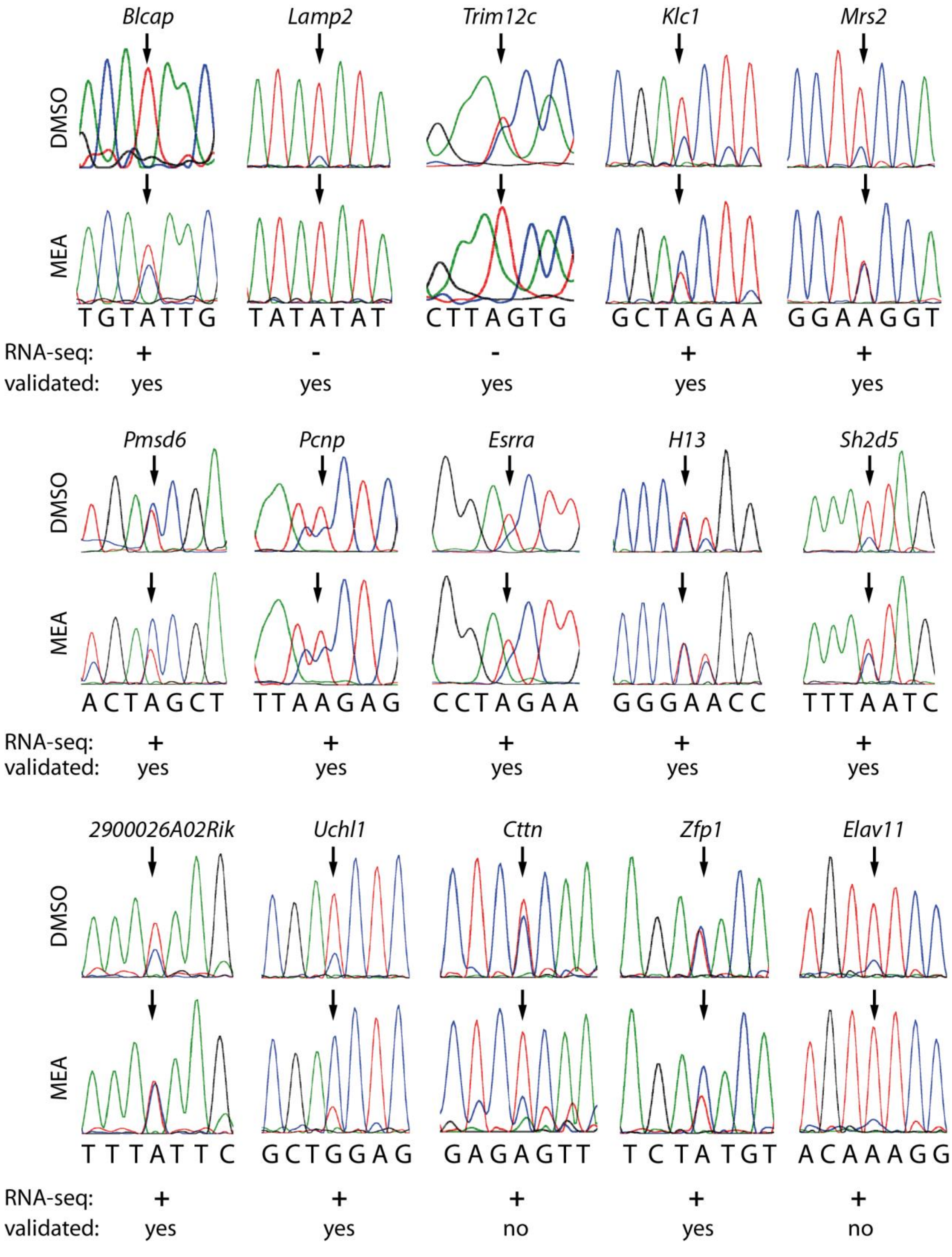

Supplemental Figure S10. Validation of changes in editing levels upon meayamycin treatment (compare to main figure 4). Sanger sequencing traces of all editing sites used for validation are shown (MEA=meayamycin; DMSO=DMSO vehicle control). The RNA-seq result is depicted below the chromatograms (RNA-seq + or - indicates an increase or decrease seen by RNA-seq). The result of the validation is shown as validated ‘yes’ or ‘no’.

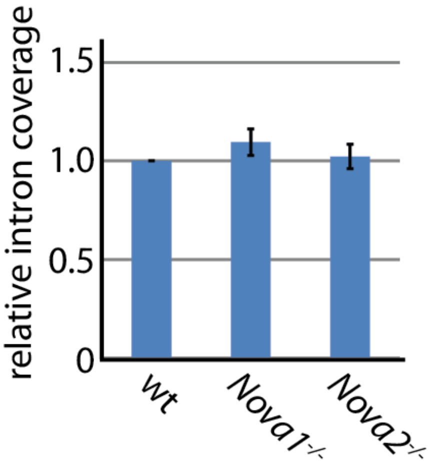

Supplemental Figure S11. Deletion of NOVA1 or NOVA2 proteins does not perturb overall splicing efficiency. Re-analysis of publicly available RNA-seq data from cortices of 6 wildtype and 3 *Nova1*$^{-/-}$ and 3 *Nova2*$^{-/-}$ mice (please compare to main figure 5). The reads have been mapped to the mouse genome (mm10). The relative intron coverage for editing sites in wildtype, *Nova1*$^{-/-}$, and *Nova2*$^{-/-}$ mice is given. Error bars=s.e.m.

Supplemental Tables S1-S8

Supplemental Table S1. Primer sequences (5' – 3') used for validation of the Nascent-seq (Nasc.) editing sites and the editing sites with significantly changed editing levels upon meayamycin (Mea) treatment.

Target	For	forward	reverse
Camk2a	Nasc.	CAACCATGGTCCCACATCCA	CCCTGCTGAACTCTGGACTG
Rims1	Nasc.	TCTGTTGCACTAAACCAGGAAA	GCTGTTCCCTCTCTCTGTTCA
Ascic2	Nasc.	TGGAAGCAGAGGGTAAAAGG	GAGCTGATGCAGAAGCAATG
Ascic2	Nasc.	CAGGACCGGAACTCAAACAT	TGTGGGTCCTGGGAATAAAA
Fam193a	Nasc.	CATCTGTCCCACCTTCTGGT	TGGCTGTCTATTGATCGATGT
Kctd8	Nasc.	TCGTCATCAGAGAGGCTTCAT	AATTCTTGCTTCAGTCTCTGTGT
Cadps2	Nasc.	ATTGGACTGAGCACCAGGTC	CTCGAGCCTTCTCAGGATGA
Mical	Nasc.	AGTGCAGCTGCTCCTTCCTA	CGGTCTCACTATGTAGCTCTGG
Usp3	Nasc.	ATAAGCCCTGGAGCTGGAGT	CCAGCTCTCACTCCCTTCTTT
Ftx	Nasc.	CATTTCAATTTAGCACGCAGA	TGGGCTGACATACTGGCATA
Blcap	Mea.	TGTGTTGACTTTTCTCTTCAGGA	CCCATGAACATGGAGTGGCT
Lamp2	Mea.	TGGGGAGGACAGATTAAATGCC	CAGTTCCCTGTTGCTCCAGA
Trim12c	Mea.	AGGGTAGAGCATCCACTGAATTAT	GGTACCTTATTCATTTGGGGTCTTT
Klc1	Mea.	GTGTGTTTCATGCCAGCCTG	ACACCTGACGCTCACTGAAG
Mrs2	Mea.	AACTAAGGGCTGTGGCTGTG	TCTGCTGTCCCAGAGAACCT
Pmsd6	Mea.	TGCTAGTGTTTTGGGGGCTT	AGCATGTATGGGCAGTGGAG
Pcnp	Mea.	ACACATAAACAGAAGGCTTTGCT	CAGACAGCTGTGAGATGCCA
Esrra	Mea.	GCTGCCTCTGTGTATCACCA	GCAGAAGCAGACAGAGCTCT
H13	Mea.	AGCAGCCTTCAAGATTCCCT	AGCTAGGTGACCCTGGGAAA
Sh2d5	Mea.	GGCAGTCCAGGGACAGATTC	TGGCTGTCCTGGAATTTGCT
2900026A02Rik	Mea.	GCAGGGACAGATGGGATCAG	TCAAGCAGGTCAGGAAGCAG
Uchl1	Mea.	CTGGGTATGGGTCAGCCTTG	GTCTGTGTGTGGGTTTGTGC
Cttn	Mea.	TTGTGCTAACTGGCCCTGAG	CCAACTCACACAAGCTGTCC
Zfp1	Mea.	ACTCAGGGGGTTAACTTTGGT	AGGTGGTGGTGCACATCTTT
Elav11	Mea.	AGGTAGAGGCAAGAGGACCA	GGAAGGGCAGTGAGTCTTCA

Supplemental Table S2. Comparison between A-to-I editing levels detected by Nascent-seq and Sanger sequencing, respectively. Genome coordinates: mm10.

Gene	Chromosome	Position	Percent A-to-I editing by nascent-seq	by Sanger-seq	Validated
Asic2	Chr11	81850116	24%	43%	Yes
Asic2	Chr11	81855010	38%	29%	Yes
Asic2	Chr11	81855013	16%	16%	Yes
Asic2	Chr11	81855050	39%	30%	Yes
Asic2	Chr11	81855086	14%	12%	Yes
Asic2	Chr11	81855102	47%	45%	Yes

Asic2	Chr11	81855103	34%	45%	Yes
Asic2	Chr11	81855116	44%	52%	Yes
Asic2	Chr11	81855182	59%	42%	Yes
Asic2	Chr11	81855188	10%	0%	No
Camk2a	Chr18	60954690	100%	55%	Yes
Fam193a	Chr5	34477808	33%	38%	Yes
Kctd8	Chr5	69271420	14%	13%	Yes
Kctd8	Chr5	69271488	47%	52%	Yes
Kctd8	Chr5	69271501	12%	12%	Yes
Cadps2	Chr6	23797919	35%	44%	Yes
Cadps2	Chr6	23797920	60%	71%	Yes
Mical	Chr6	120964212	16%	11%	Yes
Usp3	Chr9	66539721	14%	17%	Yes
Ftx	ChrX	103582512	25%	26%	Yes
Ftx	ChrX	103582515	55%	57%	Yes
Ftx	ChrX	103582533	11%	13%	Yes

Supplemental Table S3. Editing sites identified by Nascent-seq. Available as separate xlsx-file.

Supplemental Table S4. Primers used to clone randomly selected editing sites and editing complementary sites into pcDNA3.1$^{(-)}$. Fw primer 1 and rev primer 1 (fw 1, rev 1) denote the primer pair used to clone the original sequence. Fw primer 2 in combination with rev primer 1 (fw 2, rev 1) and vice versa was used to amplify the DNA without ECS. No 1 and No 2: restriction enzymes used.

Gene	Primers	Sequences	No 1	No 2
Armc9	fw 1	ATATTCTAGACCTGGGCTTCTTGCTGAGAA	XbaI	KpnI
	rev 1	ATATGGTACCAGATGGGAGAGAGGTTGCCT		
	fw 2	TTCCCCAAGTGACATTTTAAGTACAAACCAGGATGTGTCAAGCAC		
	rev 2	GTGCTTGACACATCCTGGTTTGTACTTAAAATGTCACTTGGGGAA		
Cep112	fw 1	ATATGAATTCACTTCAGATGAACAACGTTACTTT	EcoRI	KpnI
	rev 1	ATATGGTACCTGGAAACAGGATAACAGAGGCC		
	fw 2	GGAAGTTTTACAAACAGCAGTTGAGTTTTACTGGGACAGTTGTAC		
	rev 2	GTACAACTGTCCCAGTAAAACTCAACTGCTGTTTGTAAAACTTCC		
Cep89	fw 1	ATATGAATTCACCTGTTCCATCTTGTGAACCT	EcoRI	KpnI
	rev 1	ATATGGTACCCCCACAAAGCCTGCAATGTT		
	fw 2	AATCTGGGTCGGGCTGGTGACGCCCTCTTCTGGAGTG		
	rev 2	CACTCCAGAAGAGGGCGTCACCAGCCCGACCCAGATT		
Chrm2	fw 1	ATATGAATTCTCTTGTAAGTTATAGACAAATGTGTGT	EcoRI	KpnI
	rev 1	ATATGGTACCGTCCACATAATTCATCAAACTTAGGA		
	fw 2	GAAGGGGTGTTTCTGAGTTAATGGCACAGAAAGAATTGTGATCTG		
	rev 2	CAGATCACAATTCTTTCTGTGCCATTAACTCAGAAACACCCCTTC		
Chst7	fw 1	ATATTCTAGATGCTAAGTGGATTGTTTCATTTTAAAA	XbaI	KpnI
	rev 1	ATATGGTACCAGCCATACACCCTAAATATGGATAT		
	fw 2	TTCAGACACACCAAAAGAGGGCCACTGAGCCATCTCACCA		

	rev 2	TGGTGAGATGGCTCAGTGGCCCTCTTTTGGTGTGTCTGAA		
Eri3	fw 1	ATATGAATTCCAACAGCTGTGTCCTTGTGC	EcoRI	KpnI
	rev 1	ATATGGTACCGCAGCAGTGTTCTCAACTGC		
	fw 2	CTTCCTGTCTAGGAAGGAACATAAGTGATGGGAACAGAGGCC		
	rev 2	GGCCTCTGTTCCCATCACTTATGTTCCTTCCTAGACAGGAAG		
Kcnh1	fw 1	ATATTCTAGATTCCCCAGACCCCGAATACT	XbaI	KpnI
	rev 1	ATATGGTACCAGGATGCTAGCTTCCTGGTT		
	fw 2	GGTGGTTATAGAAAAGTCAACACTAAGCACACACAGGCCTCCAG		
	rev 2	CTGGAGGCCTGTGTGTGCTTAGTGTTGACTTTTCTATAACCACC		
Raf1	fw 1	ATATGAATTCGACTCAAGAGACGTGGCCAA	EcoRI	KpnI
	rev 1	ATATGGTACCTGCTGAGTGAATCACGTGTT		
	fw 2	CTTTAATCCCAGCACTCGGTCTCGAAAAAACCAAAAAAAAAAAAAAA		
	rev 2	TTTTTTTTTTTTTTTGGTTTTTTCGAGACCGAGTGCTGGGATTAAAG		
Rapgef4	fw 1	ATATGAATTCTGCTAAGGAGTAGAGACTTGGAGA	EcoRI	KpnI
	rev 1	ATATGGTACCACGCAACGTCTCTATTTTGGAG		
	fw 2	GATCTGAGATCCTCTGGGACAGCCATCCAACTCTCCAACC		
	rev 2	GGTTGGAGAGTTGGATGGCTGTCCCAGAGGATCTCAGATC		
Snx19	fw 1	ATATCTCGAGGGTGGCAGCTGCAGATAGAT	XhoI	KpnI
	rev 1	ATATGGTACCCTCCTACAGACCCACCTCGA		
	fw 2	TAGAGAGTAGACACTTCTTGCGAAATTGGTAAATGTCATGGC		
	rev 2	GCCATGACATTTACCAATTTCGCAAGAAGTGTCTACTCTCTA		

Supplemental Table S5. Oligonucleotides used to amplify and sequence the editing sites in the test constructs used to validate the accuracy of the ECS prediction. Please note that some primers match to the vector sequence and are common to several targets. Fw/rev primer=oligonucleotides used to amplify the sequence. Sanger=primer used for Sanger sequencing.

Gene	fw primer	rev primer	Sanger
Kcnh1	ATCCCAACCAGGAGGCTAGT	GCTGATCAGCGGTTTAAACTT	GGTGGTTATAGAAAAGTCAACACTCC
Armc9	CAGGTCAGAGCCAGGAGACA	GCTGATCAGCGGTTTAAACTT	TCTGGGAAGGCATTCAATTTGT
Cep112	ACCAGAGGCTCAACACATGG	GCTGATCAGCGGTTTAAACTT	TCTGGGAAGGCATTCAATTTGT
Eri3	CGGCATGGACACTGGAGAG	GCTGATCAGCGGTTTAAACTT	GCCACCTTTCCAGCTCTTTC
Chst7	GGAGACCCAAGCTGGCTAG	GTGTACGCCAGAAAAAGTGTCA	GTGTACGCCAGAAAAAGTGTCA
Snx19	GGAGACCCAAGCTGGCTAG	TGTGTGTGTGTATGTATGTGAAGA	TGTGTGTGTGTATGTATGTGAAGA
Cep89	GGAGACCCAAGCTGGCTAG	TGCTCTTAAAAATCCAAAGTCCAGG	TGCTCTTAAAAATCCAAAGTCCAGG
Rappgef4	GGAGACCCAAGCTGGCTAG	TCATCCGATTCCCTGGAACTG	TCATCCGATTCCCTGGAACTG
Raf1	AGCAGCACAGCATTGATCCT	AAAGTGCACGCCTTTAAGCC	AAAGTGCACGCCTTTAAGCC
Chrm2	AGTGCTGTTACAAATGCCTAAAAGT	GCTGATCAGCGGTTTAAACTT	AGTGCTGTTACAAATGCCTAAAAGT

Supplemental Table S6. Editing sites identified in bone marrow and primary neurons plus changes in editing levels upon splicing inhibition. Available as separate xlsx-file.

Supplemental Table S7. Editing sites identified in *Nova1*$^{-/-}$ and *Nova2*$^{-/-}$ knockout mice plus changes in editing levels as compared to wildtype. Available as separate xlsx-file.

Supplemental Table S8. Best evidence and a reference for the location of editing complementary sites (ECS) for the editing targets used in main figure 6 are listed. The structure prediction has been done using RNAfold and visualized using forna. All structure predictions are shown in Supplemental Dataset S2. We define the ECS_type as intronic if the editing-competent double-stranded RNA is formed between the edited exon and the downstream or upstream intron. Vice versa we call the ECS_type exonic if the editing-competent stem is formed within the edited exon. Genome coordinates: hg19.

Gene	Chr.	Coordinate	ECS_type	Best evidence	Reference
Best1	Chr11	61724916	intronic	structure prediction	this study
Flna	ChrX	153579950	intronic	mutational analysis	(Jain et al. 2018)
Flnb	Chr3	58141801	intronic	mutational analysis	Pullirsch et al. (unpublished)
Cog3	Chr13	46090371	intronic	structure prediction	this study
Copa	Chr1	160302244	intronic	structure prediction	this study
Gria2	Chr4	158257875	intronic	mutational analysis	(Higuchi et al. 1993)
Gria2	Chr4	158257879	intronic	mutational analysis	(Higuchi et al. 1993)
Gria2	Chr4	158281294	intronic	mutational analysis	(Lomeli et al. 1994)
Gria3	ChrX	122598962	intronic	mutational analysis	(Lomeli et al. 1994)
Gria4	Chr11	105804694	intronic	mutational analysis	(Lomeli et al. 1994)
Grik1	Chr21	30953750	intronic	mutational analysis	(Herb et al. 1996)
Grik2	Chr6	102372572	intronic	mutational analysis	(Herb et al. 1996)
Grik2	Chr6	102372589	intronic	mutational analysis	(Herb et al. 1996)
Neil1	Chr15	75646087	intronic	mutational analysis	(Yeo et al. 2010)
Neil1	Chr15	75646086	intronic	mutational analysis	(Yeo et al. 2010)
Tmem63b	Chr6	44120349	intronic	structure prediction	this study
Azin1	Chr8	103841636	exonic	structure prediction	this study
Blcap	Chr20	36147563	exonic	structure prediction	this study
Cacna1d	Chr3	53820892	exonic	structure prediction	this study
Cadps	Chr3	62423807	exonic	structure prediction	this study
Cdk13	Chr7	39990302	exonic	structure prediction	(Maas et al. 2011); this study
Dcaf16	Chr4	17805279	exonic	structure prediction	this study
Elfn2	Chr22	37770174	exonic	structure prediction	this study
Gabra3	ChrX	151358319	exonic	mutational analysis	(Ohlson et al. 2007)
Gipc1	Chr19	14593693	exonic	structure prediction	this study
Grm4	Chr6	34100903	exonic	structure prediction	this study
Igfbp7	Chr4	57976234	exonic	structure prediction	(Gommans et al. 2008); this study
Igfbp7	Chr4	57976286	exonic	structure prediction	(Gommans et al. 2008); this study
Kcna1	Chr12	5021742	exonic	mutational analysis	(Bhalla et al. 2004); this study
Kcnma1	Chr10	79397298	exonic	structure prediction	this study
Mll4	Chr19	36211399	exonic	structure prediction	this study
Myo19	Chr17	34866719	exonic	structure prediction	this study
Nova1	Chr14	26917515	exonic	structure prediction	(Irimia et al. 2012); this study
Osgep	Chr14	20920211	exonic	structure prediction	this study
Pdcd7	Chr15	65425319	exonic	structure prediction	this study
Pdcd7	Chr15	65425334	exonic	structure prediction	this study

Pdcd7	Chr15	65425992	exonic	structure prediction	this study
Pdcd7	Chr15	65426067	exonic	structure prediction	this study
Plch2	Chr1	2436080	exonic	structure prediction	this study
Sh3bp2	Chr4	2835556	exonic	structure prediction	this study
Son	Chr21	34923319	exonic	structure prediction	this study
Son	Chr21	34924105	exonic	structure prediction	this study
Son	Chr21	34922801	exonic	structure prediction	this study
Ttll3	Chr3	9876560	exonic	structure prediction	this study
Unc80	Chr2	210835613	exonic	structure prediction	this study

Supplemental Materials and Methods

Experimental validation of editing complementary site (ECS) predictions

A DNA sequence encompassing the randomly selected editing site and the predicted ECS was amplified from genomic mouse DNA and cloned into pcDNA3.1$^{(-)}$ using primers fw 1 and rev 1 (Supplemental Table S4). To remove the ECS primers fw 1/rev 2 and fw 2/rev 1 were used, respectively. Subsequently, both PCR products were combined and again amplified with primers fw 1 and rev 1. Restriction enzymes were used as indicated (Supplemental Table S4). To test editing, 3x10^{5} Hek293 cells were plated per well using 6-well dishes. 24 h after plating, 0.5 µg of plasmid (plus or minus ECS) was co-transfected with 3.5 µg of a plasmid expressing Flag-rADAR2 using poly-ethyleneimine (Polysciences cat# 23966). 2 days after transfection RNA was isolated using Trifast™ (Peqlab cat# 30-2010) following the manufacturer's instructions. Subsequently, RNA was DNase I treated (New England Biolabs, cat# M0303). Following reverse transcription with random hexamers (Integrated DNA Technologies) and M-MuLV reverse transcriptase (New England Biolabs, cat# M0253) the editing site was PCR amplified using OneTaq Green PCR mix (New England Biolabs, cat# M0482) and primer pairs as indicated (Supplemental Table S5). Following gel extraction using Monarch® DNA Gel Extraction Kit (New England Biolabs, cat# T1020) the PCR product was submitted to Sanger sequencing (Eurofins Genomics) using the primer as indicated (Supplemental Table S5). If not stated otherwise all custom primers were obtained from Microsynth Austria.

Supplemental Datasets

Supplemental Dataset S1: Structure predictions to validate bioinformatically predicted ECS. The structure has been predicted using RNAfold and visualized using forna (Lorenz et al. 2011; Kerpedjiev et al. 2015). The editing site is shown in red; the editing complementary site is highlighted in blue. Light blue indicates the sequence that has been deleted in the deltaECS constructs.

Transcript: *Armc9*

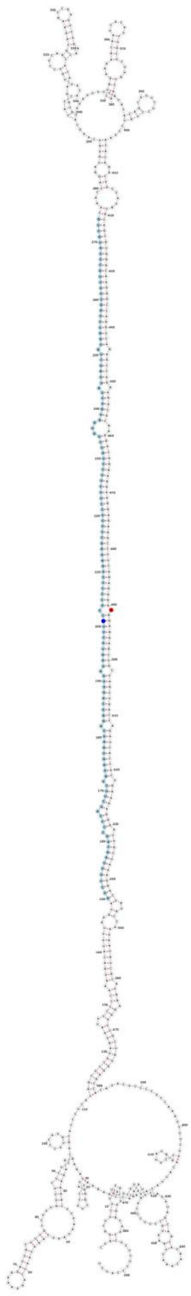

Transcript: *Cep89*

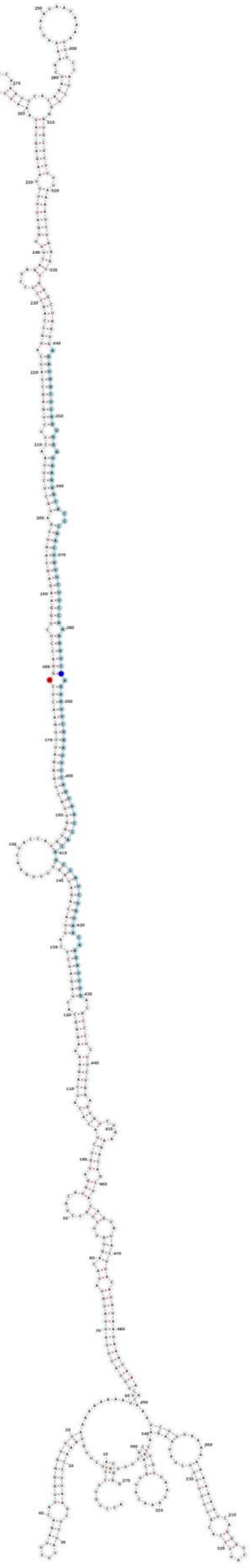

Transcript: *Cep112*

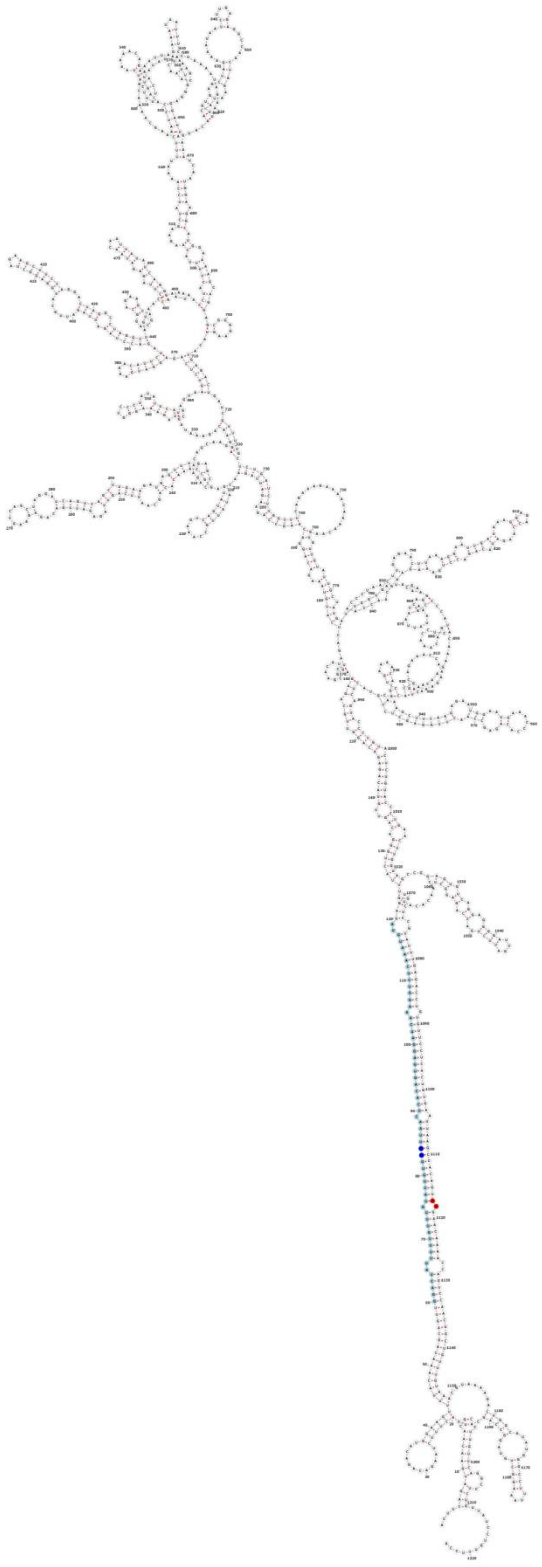

Transcript: *Chrm2*

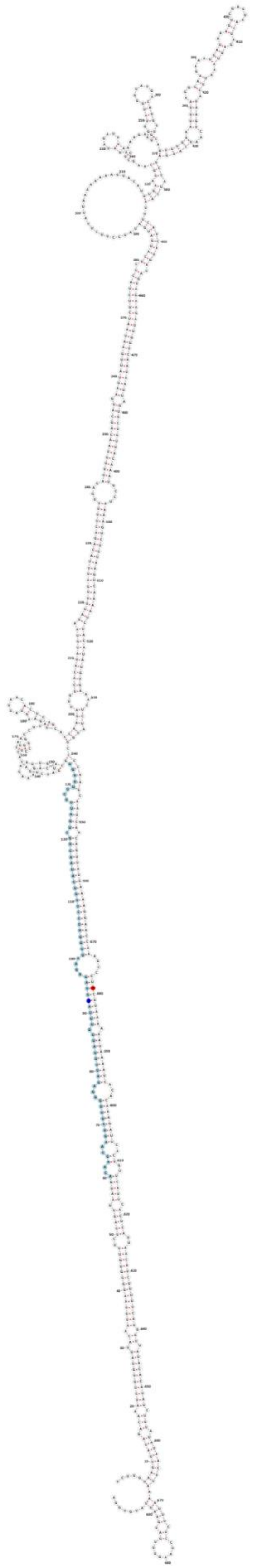

Transcript: *Chst7*

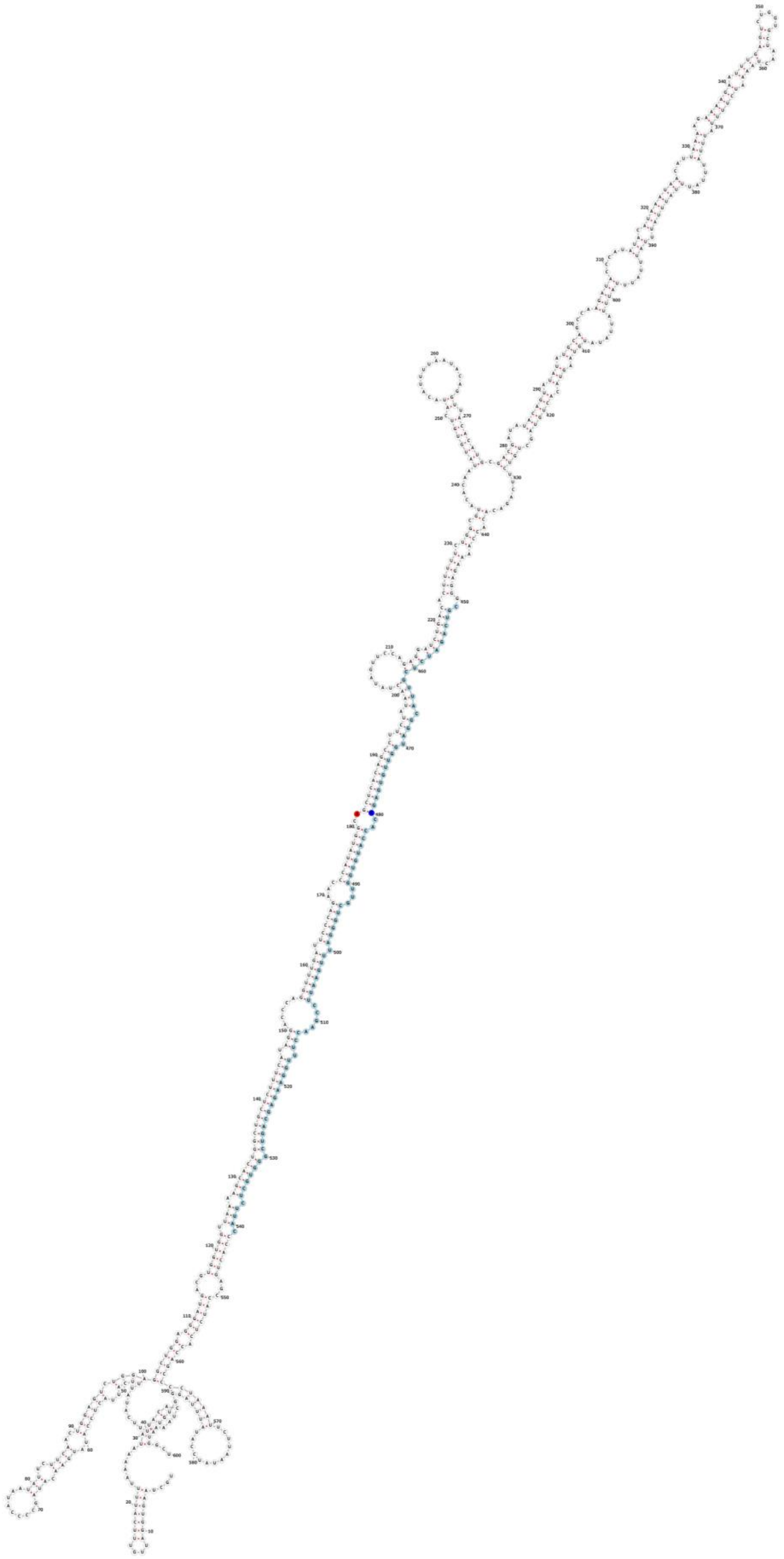

Transcript: *Eri3*

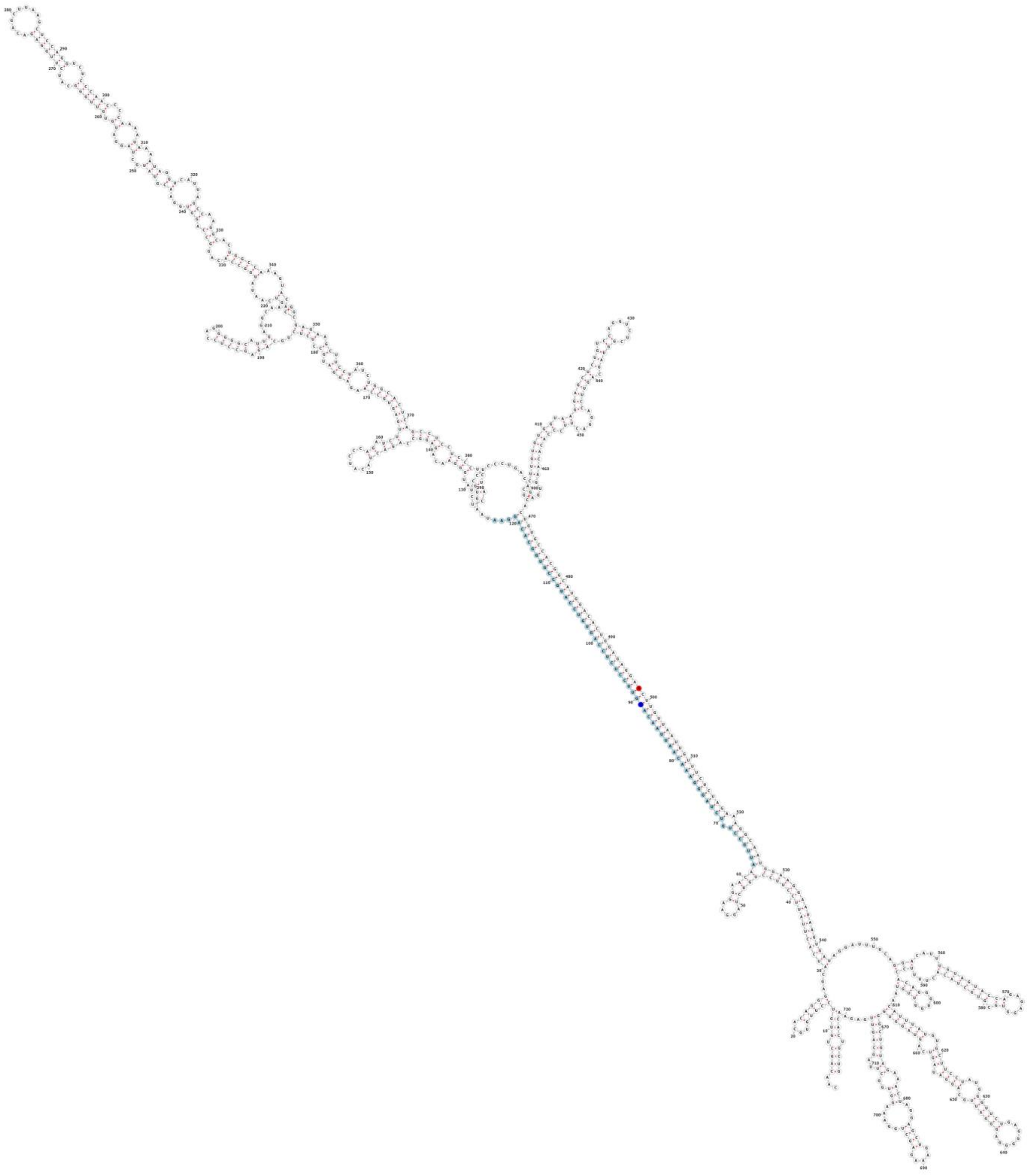

Transcript: *Kcnh1*

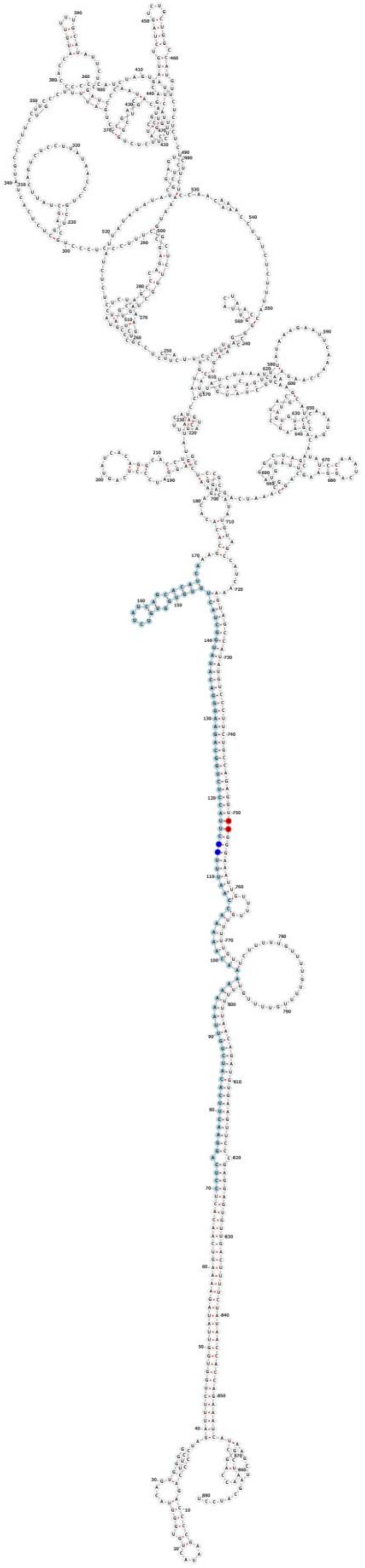

Transcript: *Rapgef4*

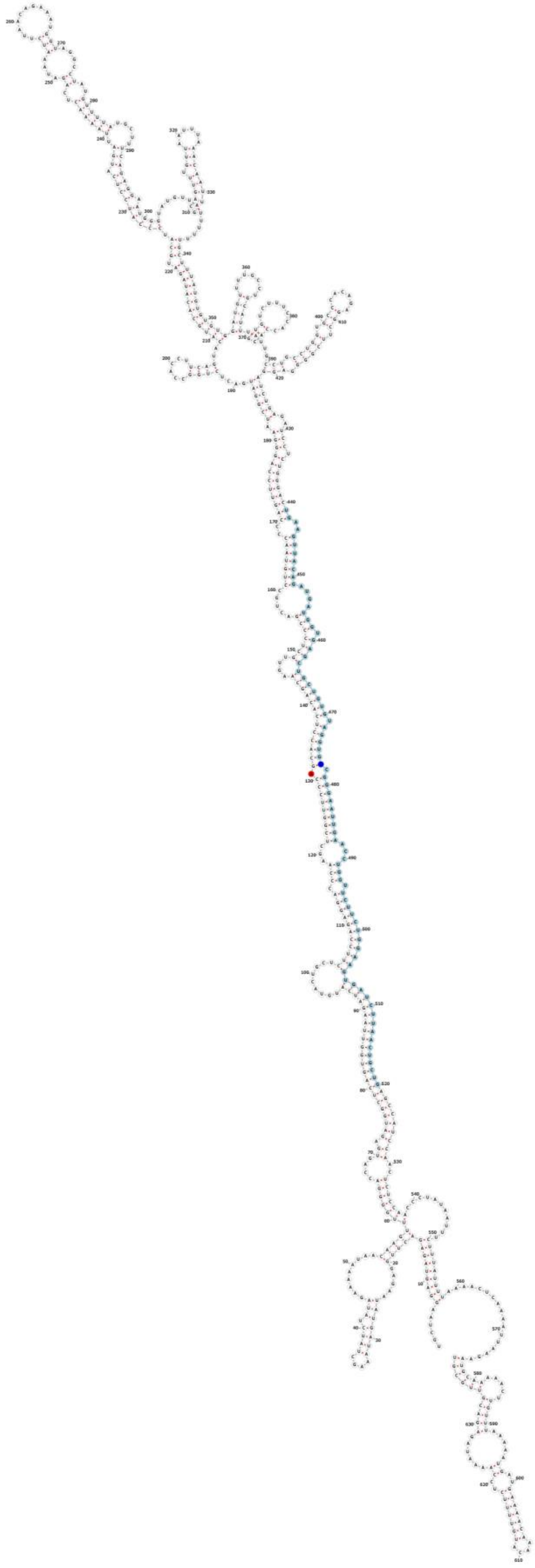

Transcript: *Snx19*

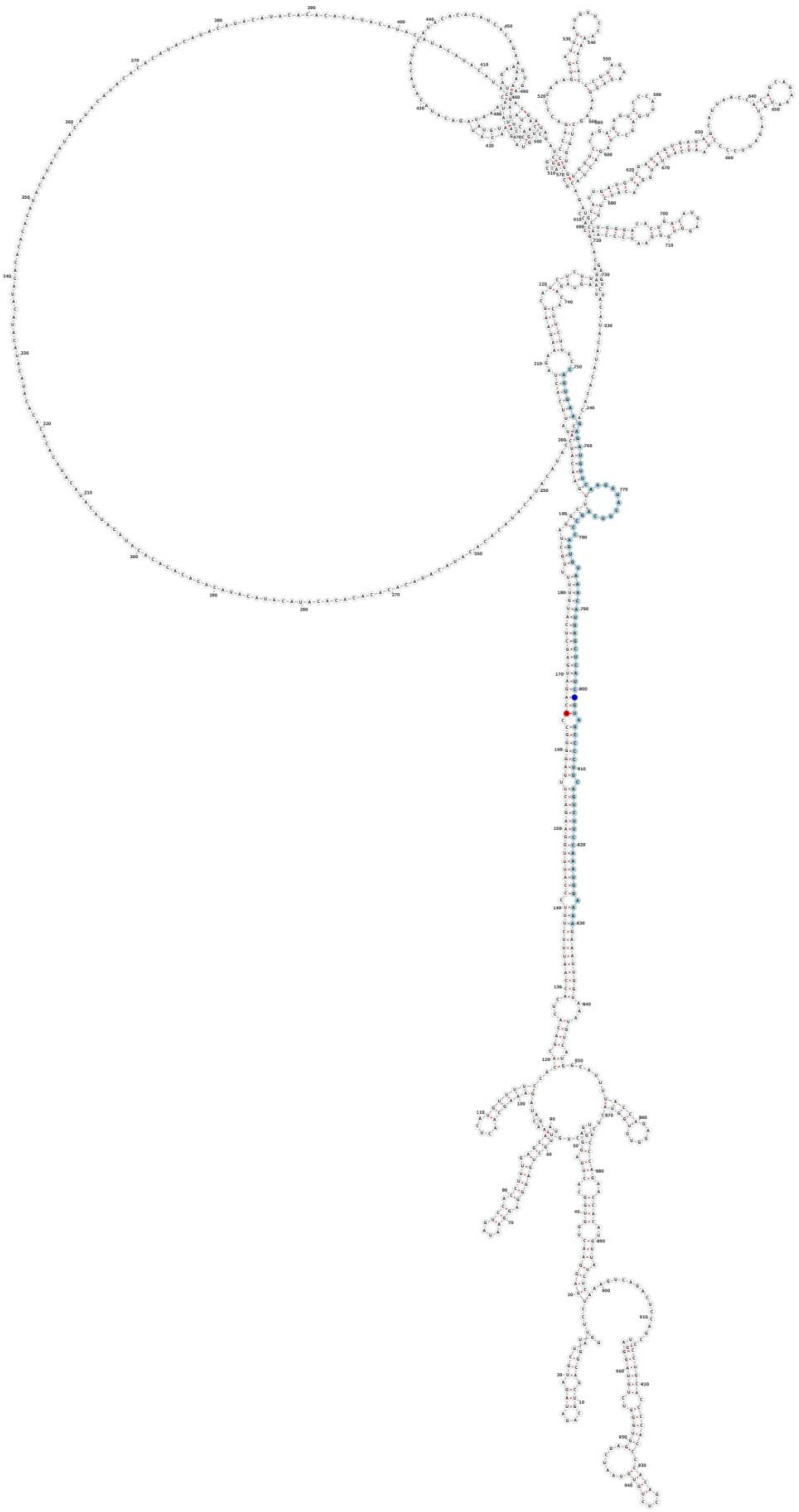

Transcript: *Tnpo3*

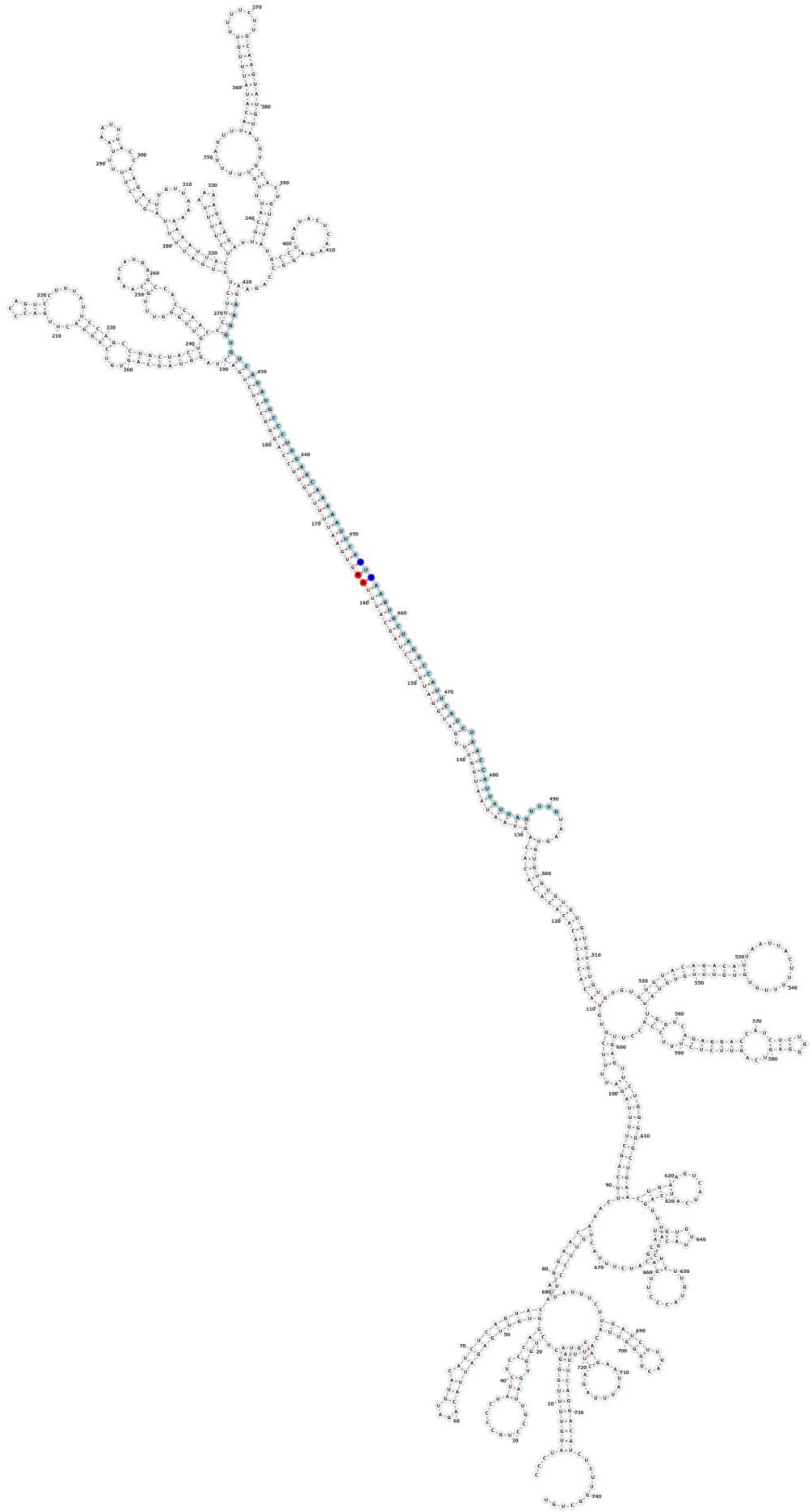

Supplemental Dataset S2. Structure predictions of all edited transcripts used in Figure 6 were no experimental evidence for the location of the editing complementary site (ECS) is available. The structure has been predicted using RNAfold and visualized using forna. The exonic sequence is highlighted in yellow. The edited adenosine is marked in red. The intronic sequence is not colored.

Transcript: *Best1*

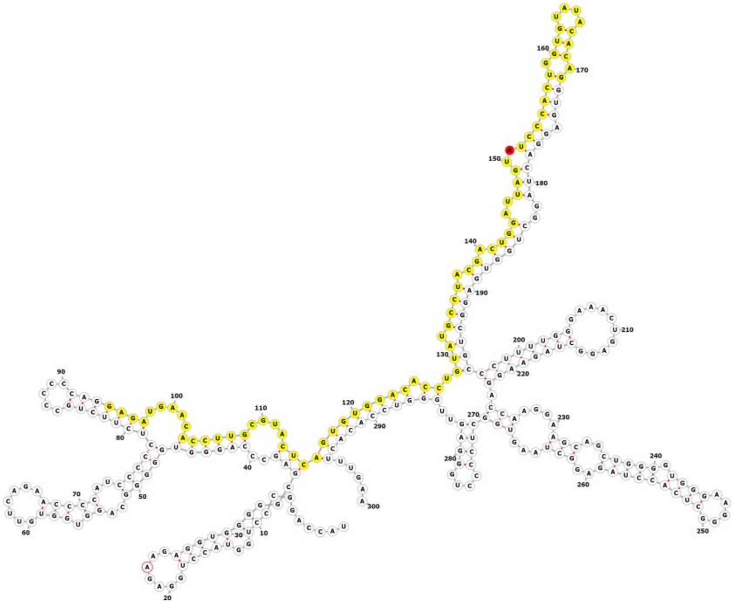

Transcript: *Cog3*

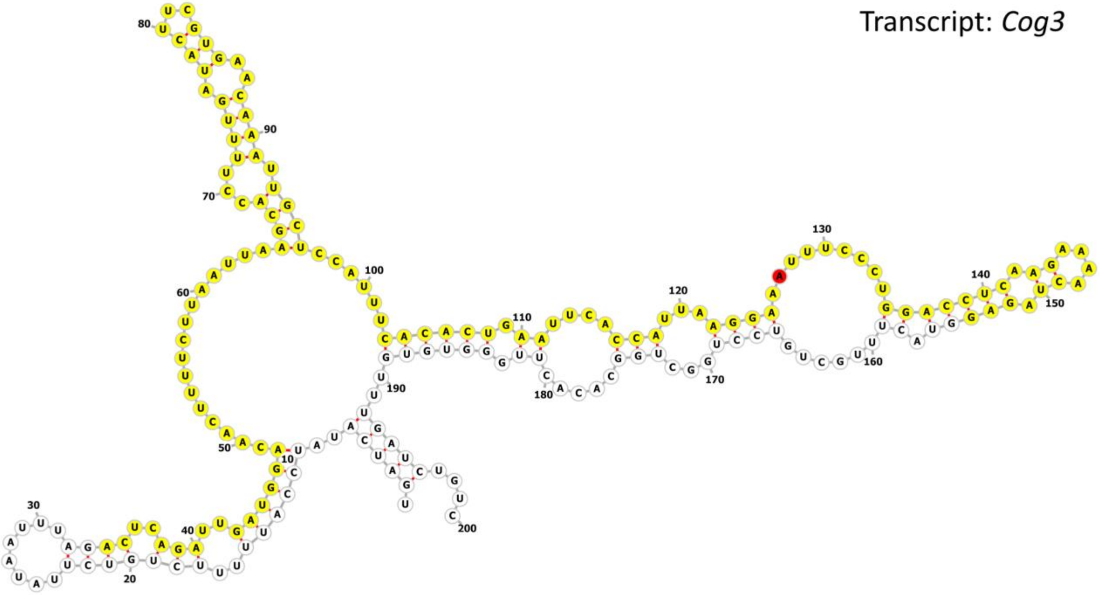

Transcript: *Copa*

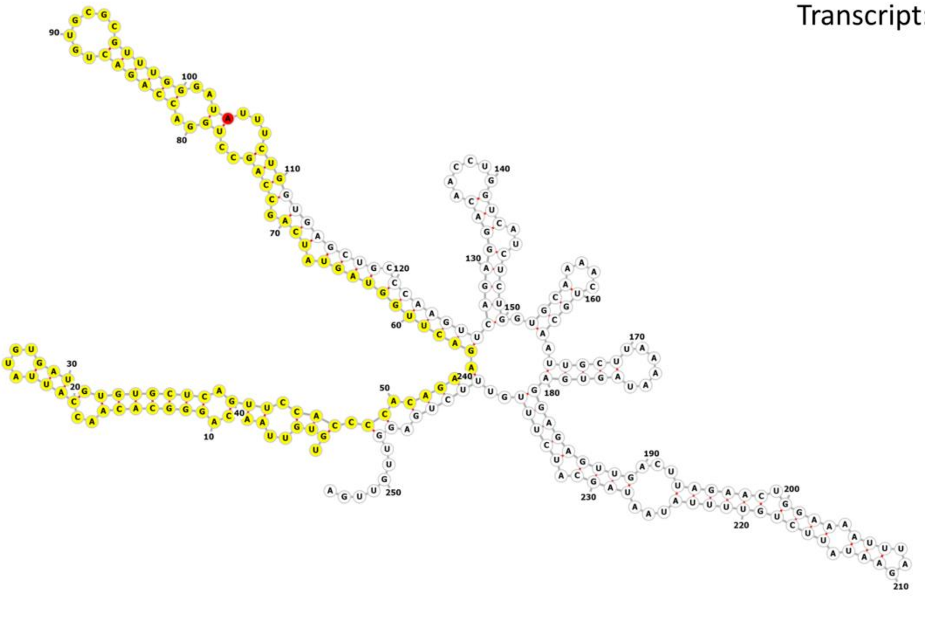

Transcript: *Tmem63b*

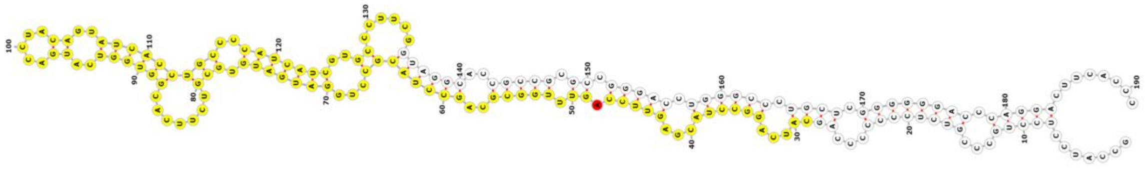

Transcript: *Azin1*

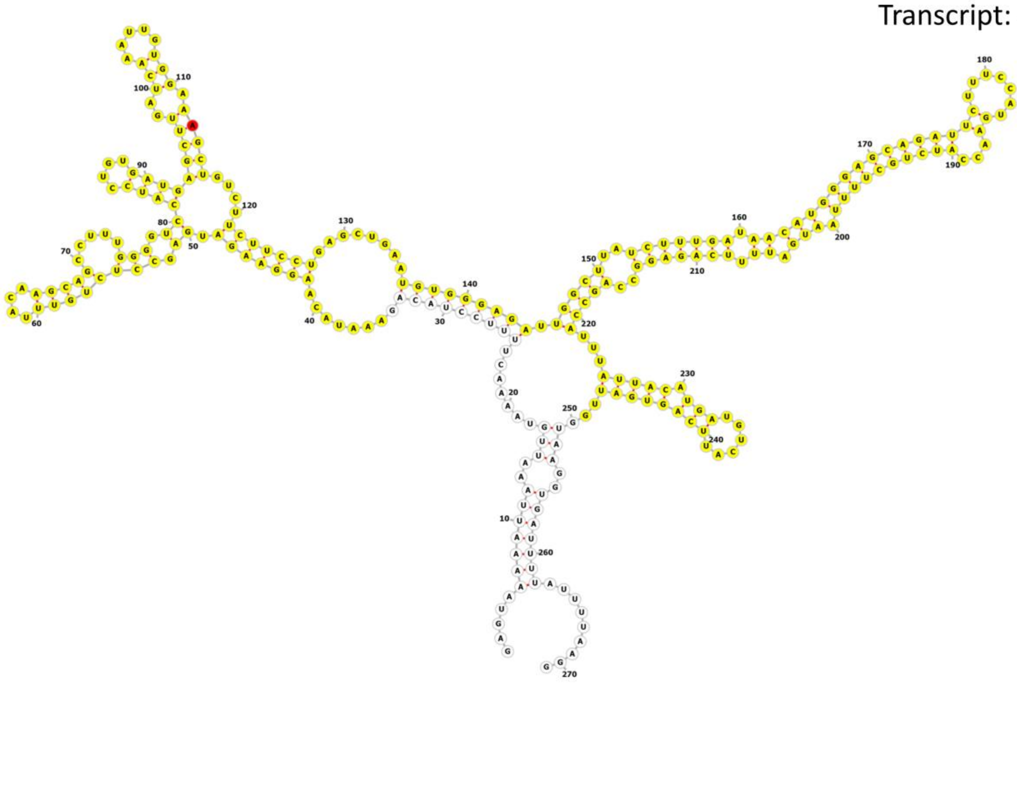

Transcript: *Blcap*

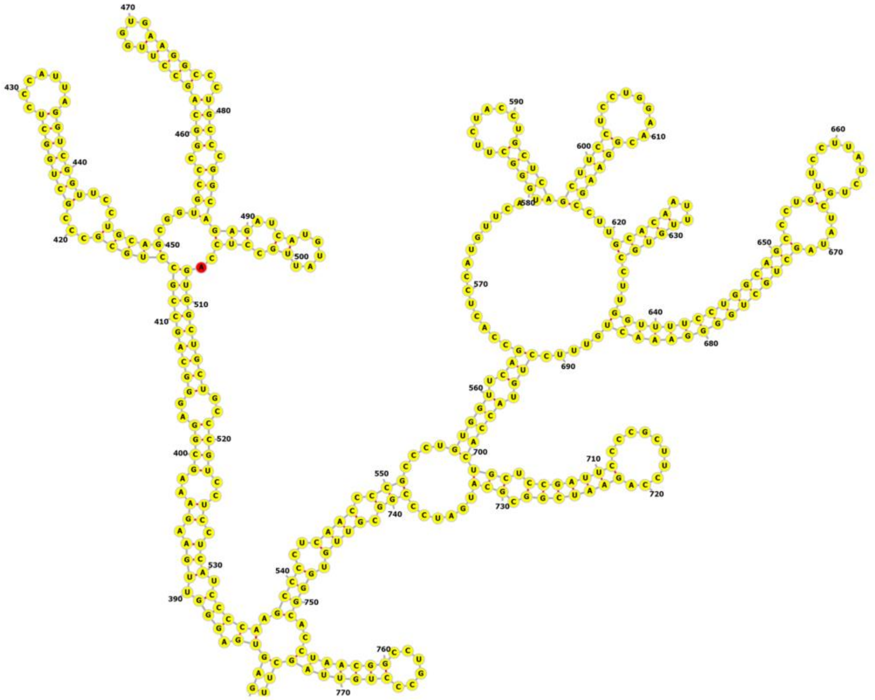

Transcript: *Cacna1d*

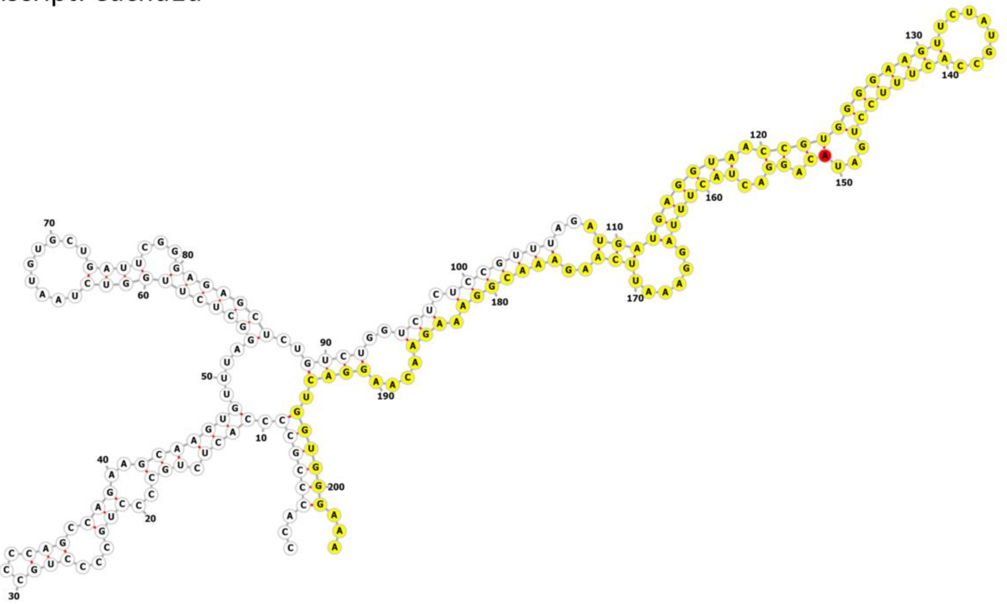

Transcript: *Cadps*

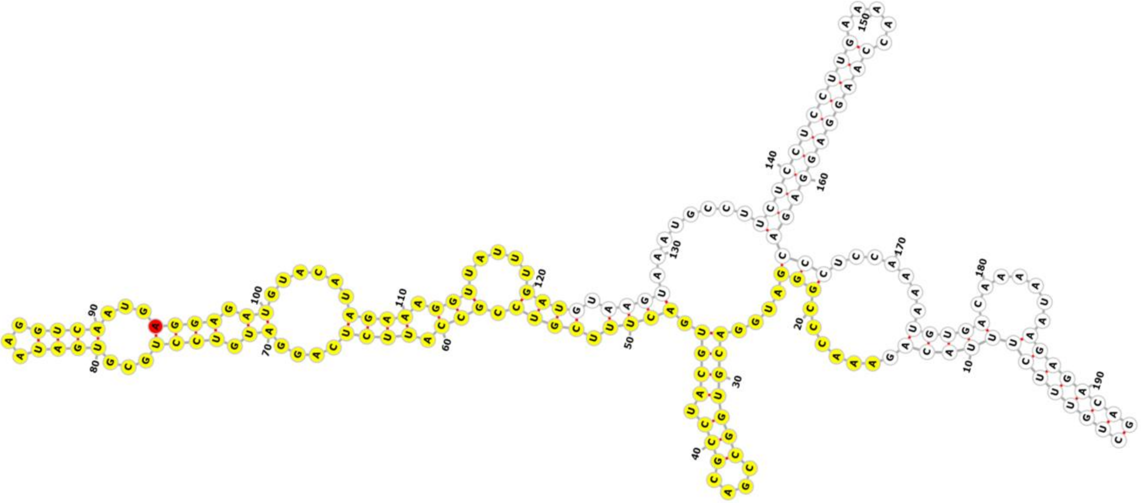

Transcript: *Cdk13*

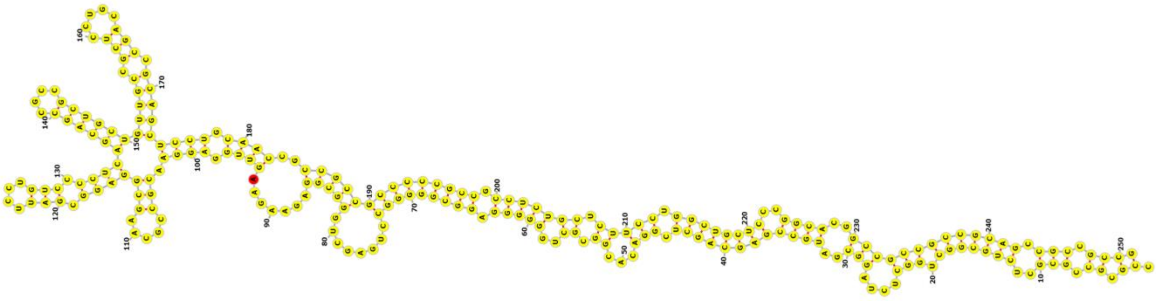

Transcript: *Dcaf16*

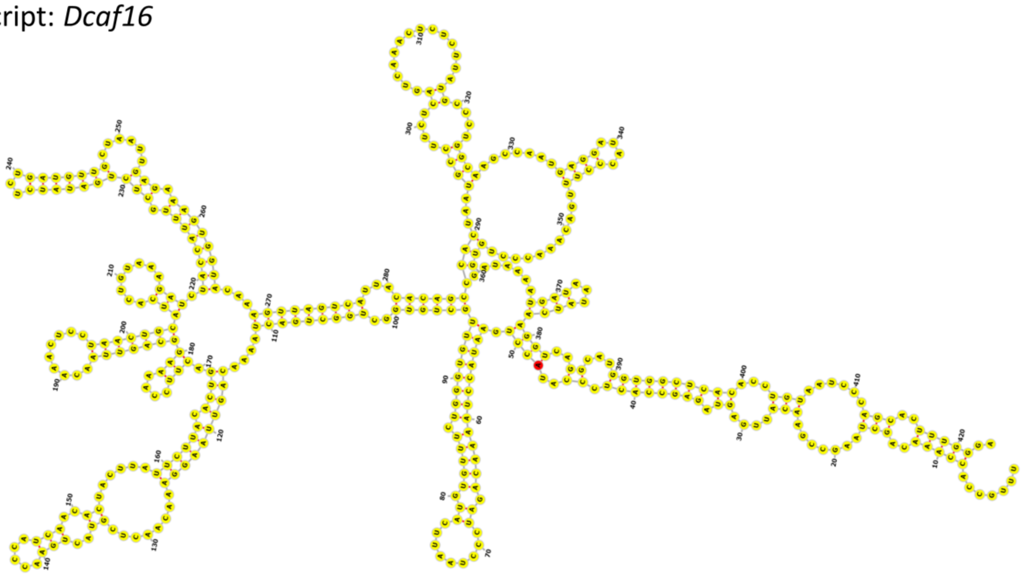

Transcript: *Elfn2*

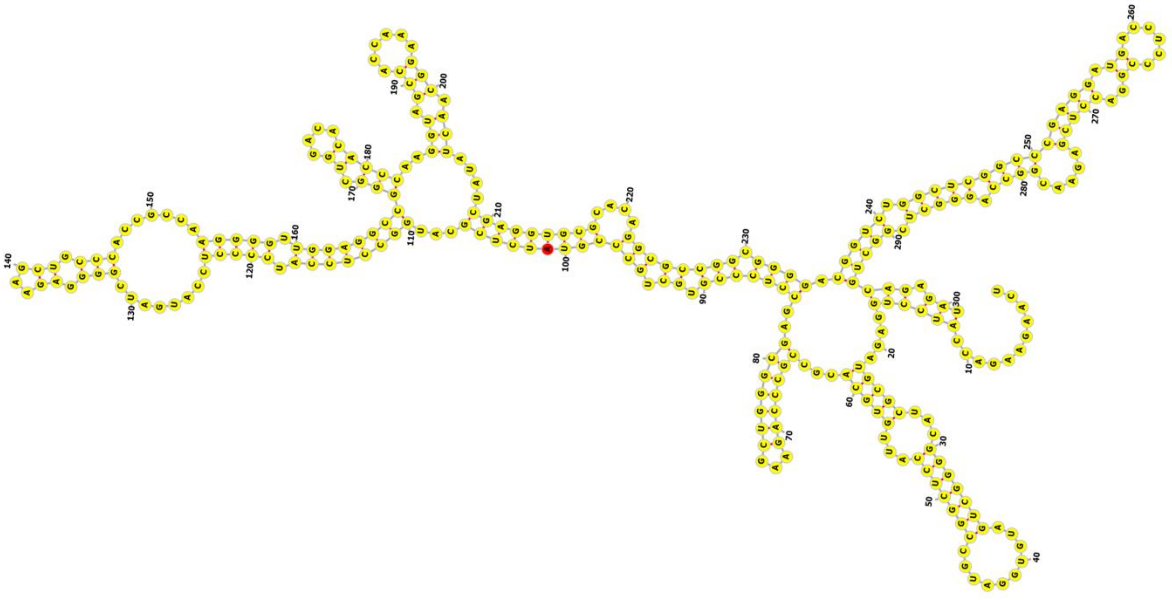

Transcript: *Gipc1*

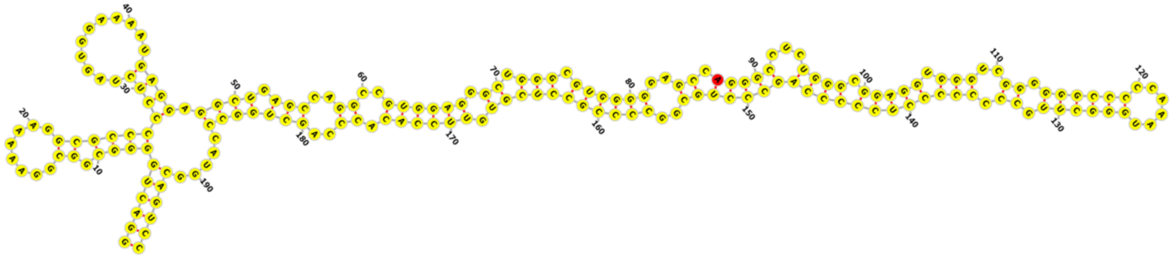

Transcript: *Grm4*

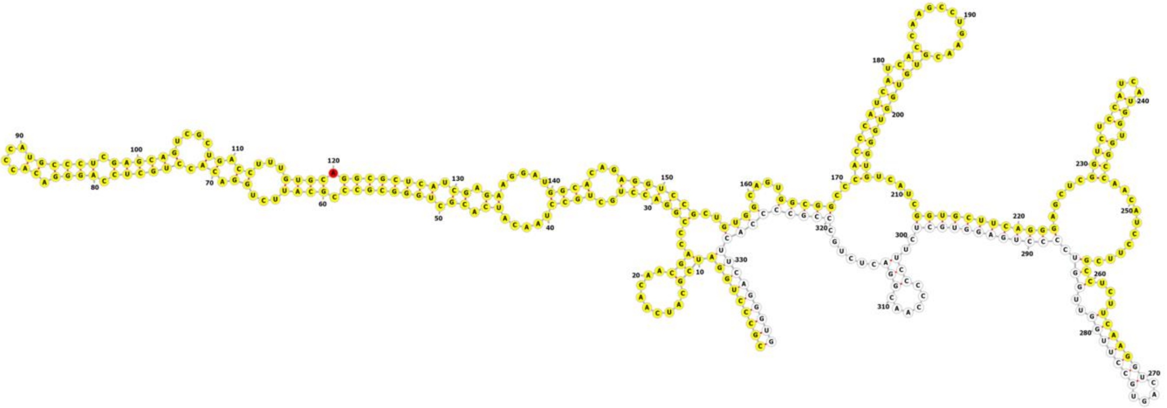

Transcript: *Igfbp7*

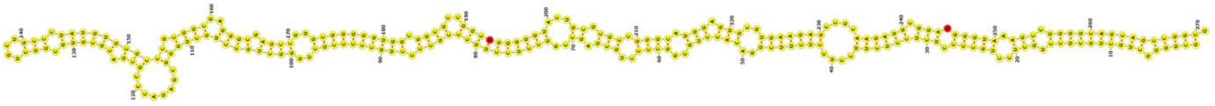

Transcript: *Kcna1*

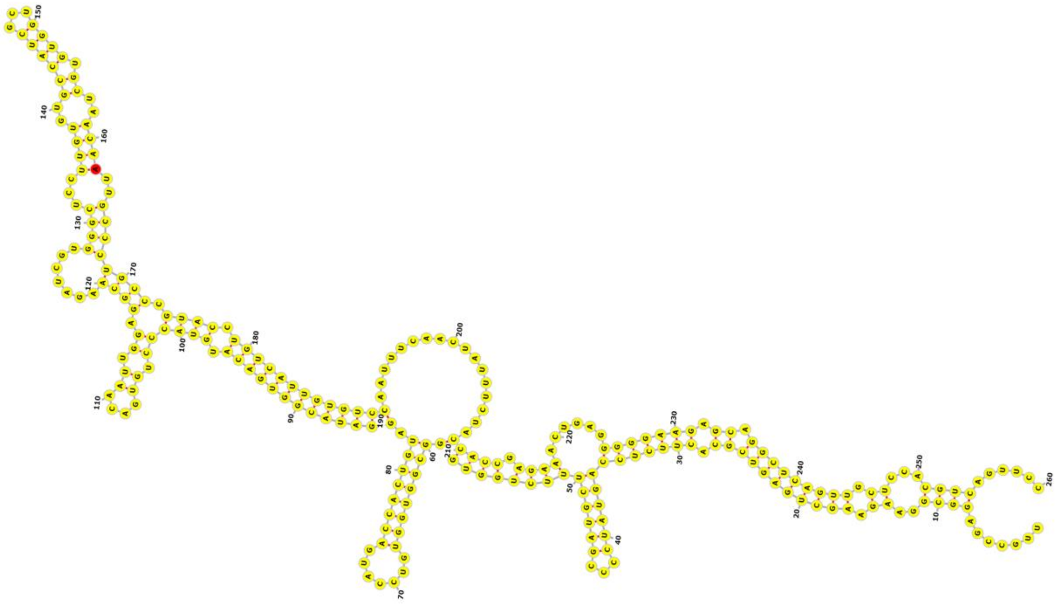

Transcript: *Kcnma1*

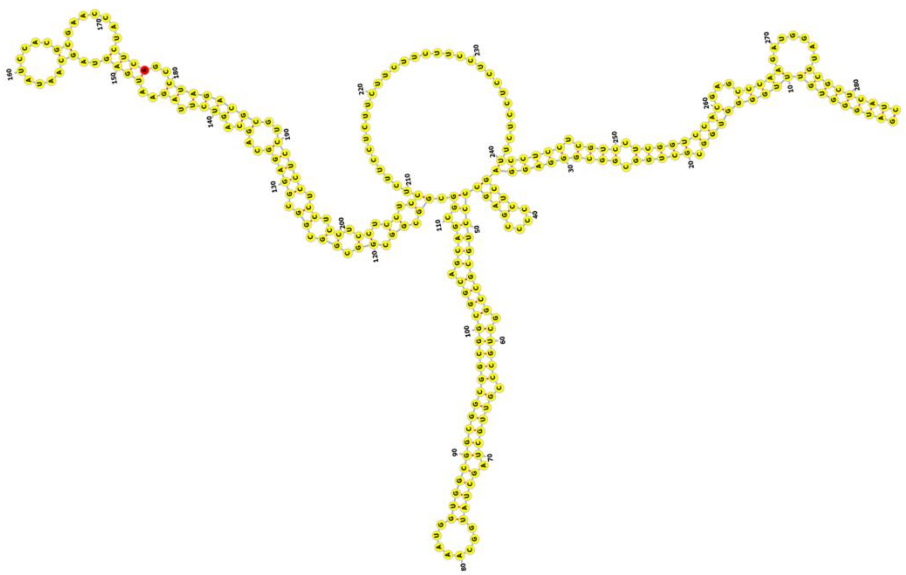

Transcript: *Mll4*

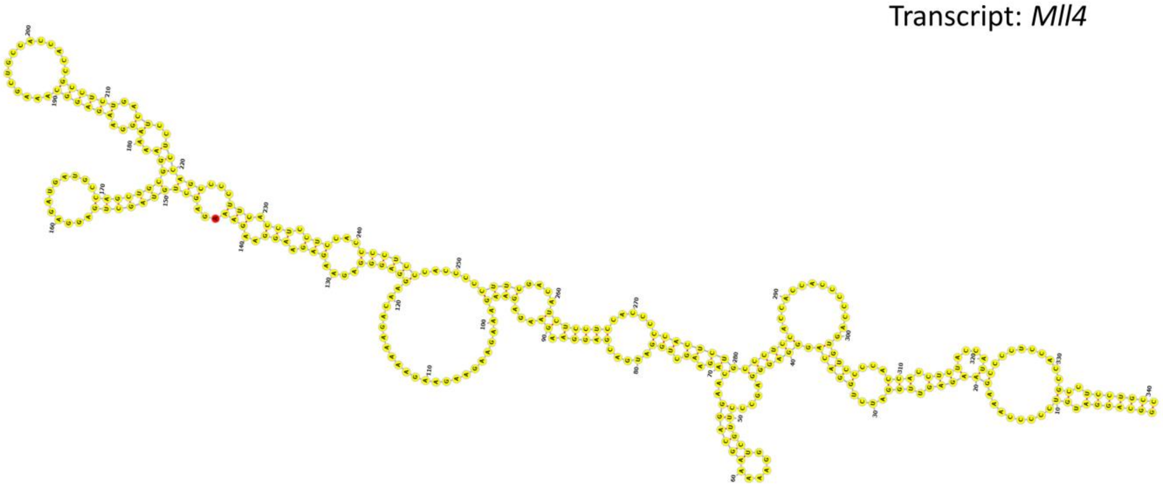

Transcript: *Myo19*

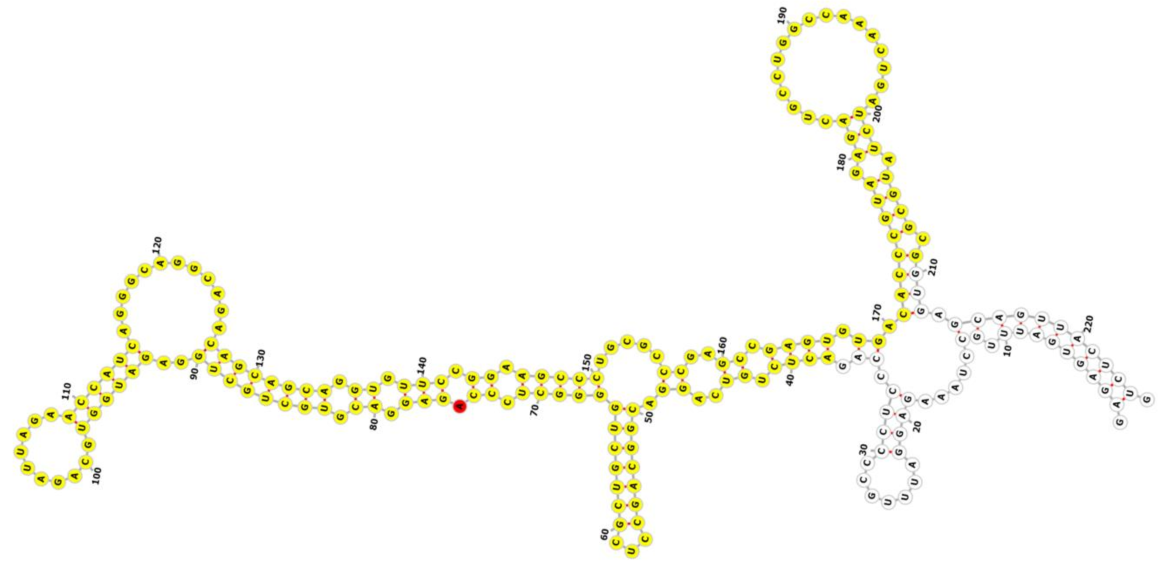

Transcript: *Nova1*

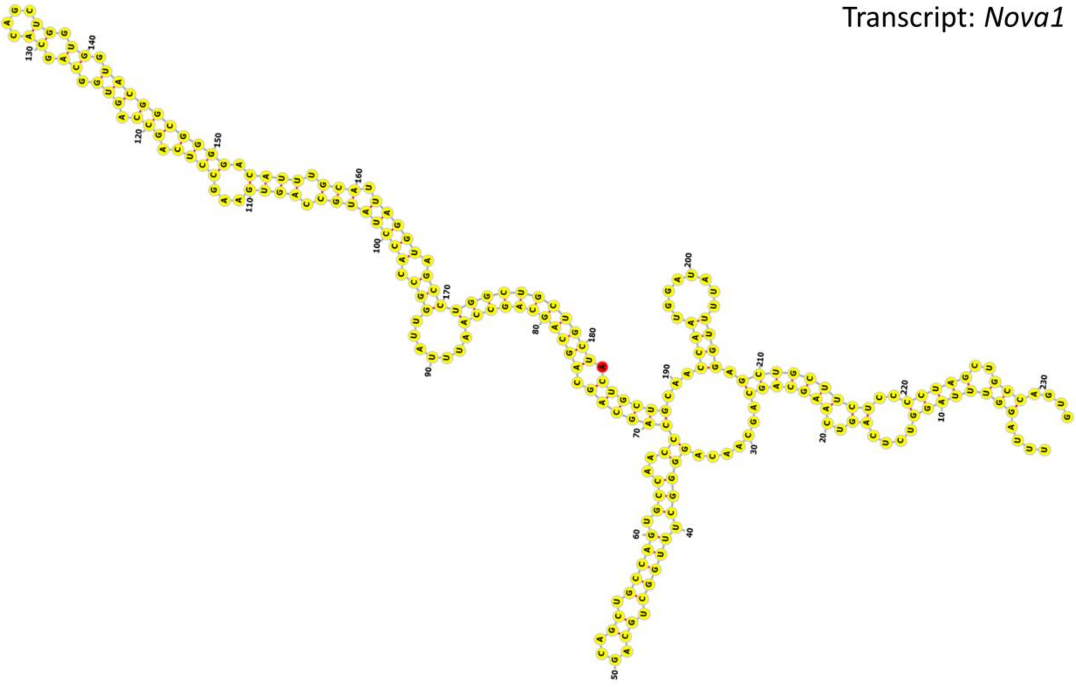

Transcript: *Osgep*

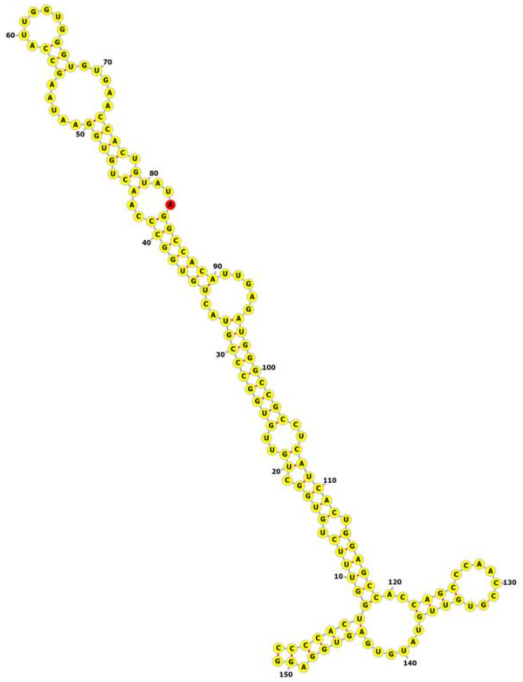

Transcript: *Pdcd7*

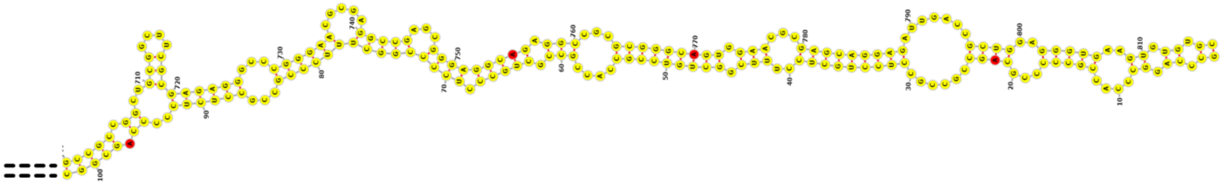

Transcript: *Plch2*

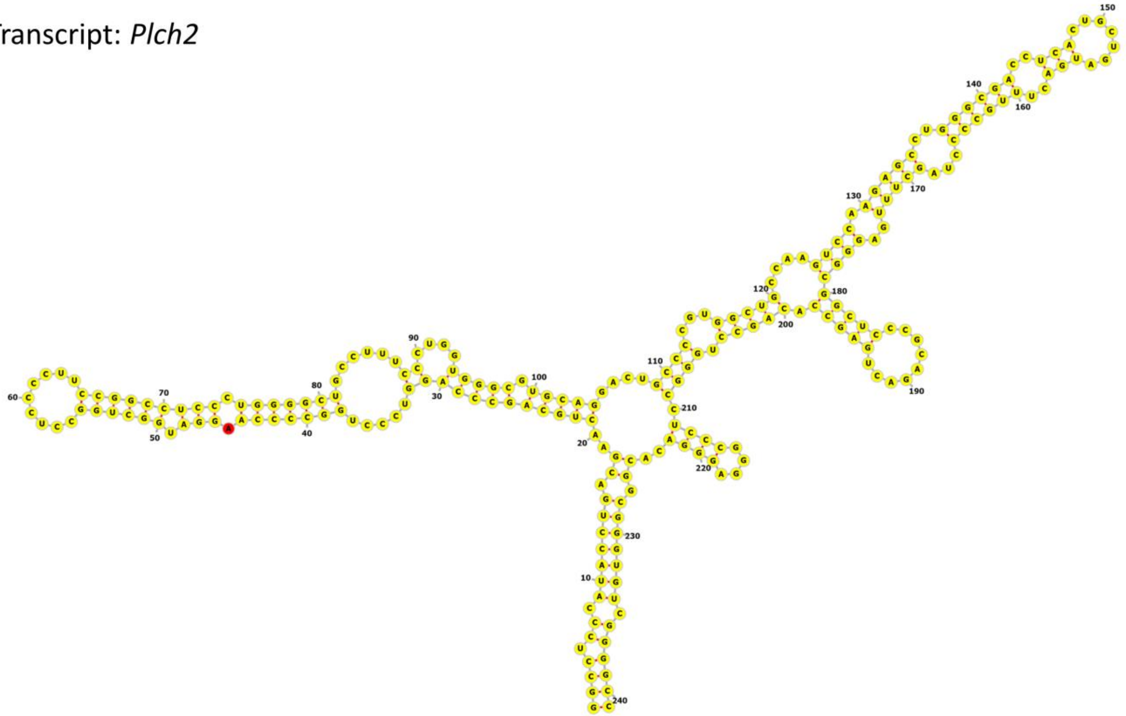

Transcript: *Sh3bp2*

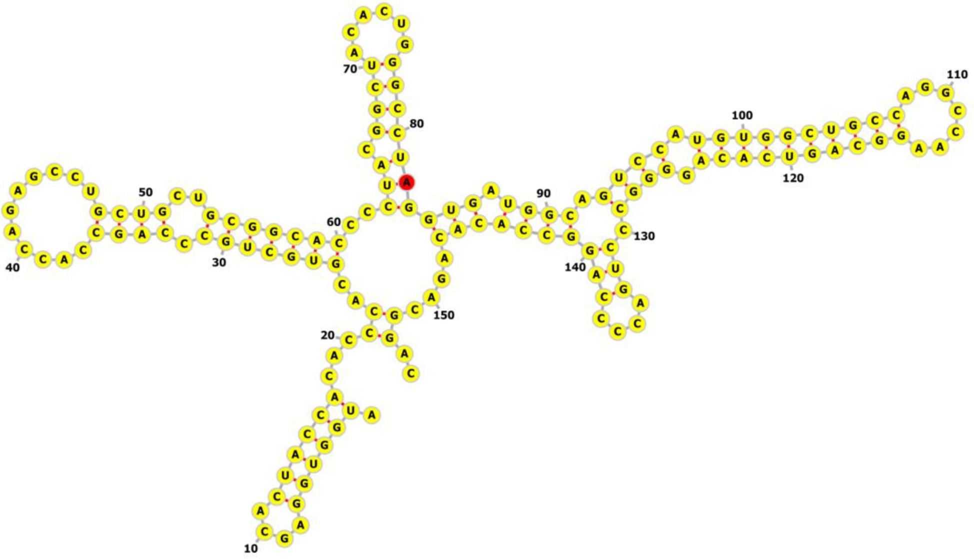

Transcript: *Son*

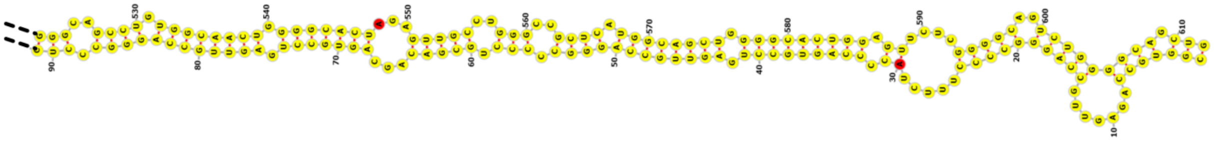

Transcript: *Son*

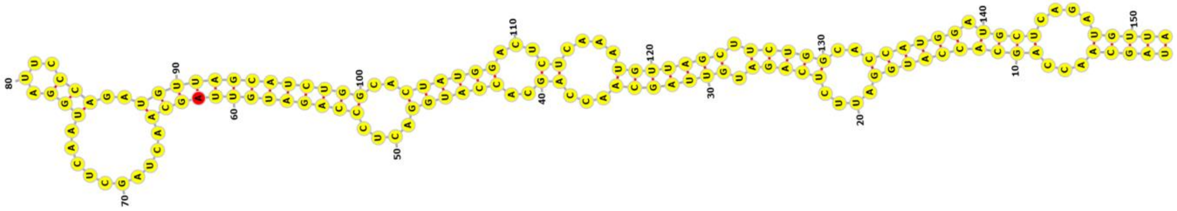

Transcript: *Tll3*

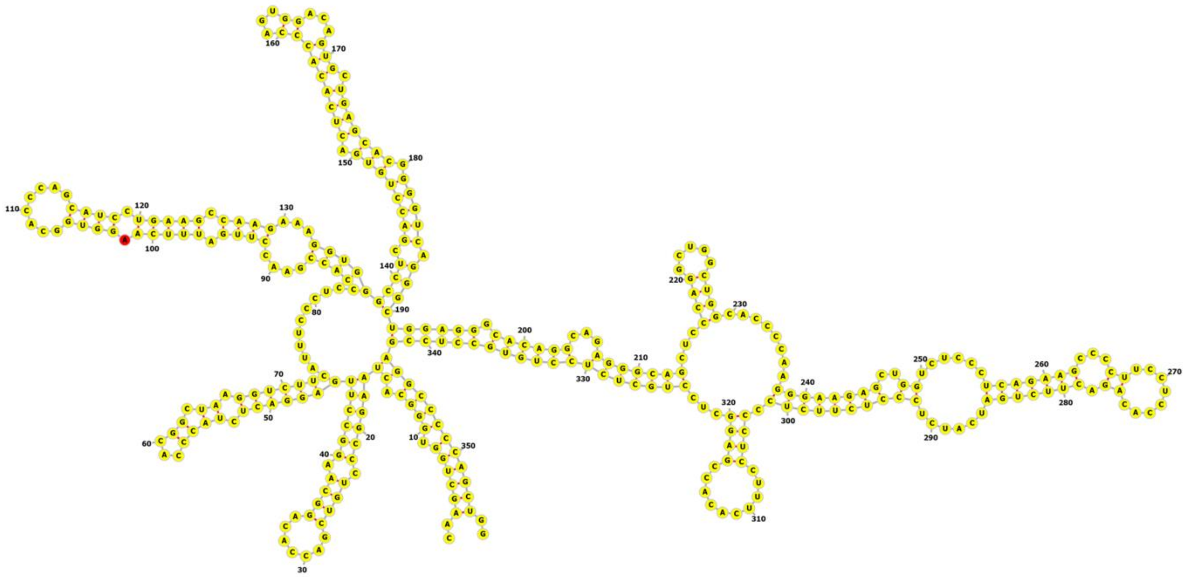

Transcript: *Unc80*

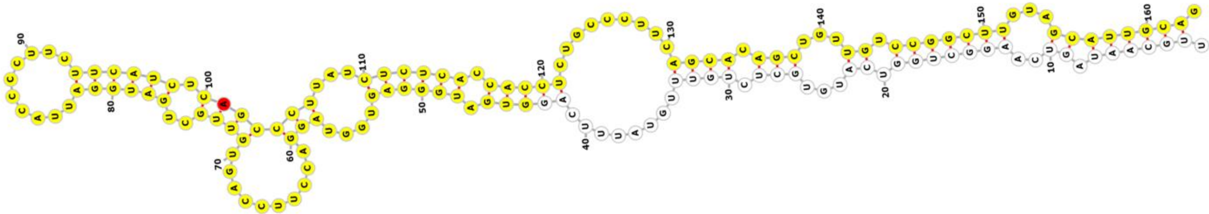

Supplemental References

Bhalla T, Rosenthal JJ, Holmgren M, Reenan R. 2004. Control of human potassium channel inactivation by editing of a small mRNA hairpin. *Nat Struct Mol Biol* **11**(10): 950-956.

Danecek P, Nellaker C, McIntyre RE, Buendia-Buendia JE, Bumpstead S, Ponting CP, Flint J, Durbin R, Keane TM, Adams DJ. 2012. High levels of RNA-editing site conservation amongst 15 laboratory mouse strains. *Genome Biol* **13**(4): 26.

Gommans WM, Tatalias NE, Sie CP, Dupuis D, Vendetti N, Smith L, Kaushal R, Maas S. 2008. Screening of human SNP database identifies recoding sites of A-to-I RNA editing. *RNA* **14**(10): 2074-2085.

Herb A, Higuchi M, Sprengel R, Seeburg PH. 1996. Q/R site editing in kainate receptor GluR5 and GluR6 pre-mRNAs requires distant intronic sequences. *Proceedings of the National Academy of Sciences of the United States of America* **93**(5): 1875-1880.

Higuchi M, Single FN, Kohler M, Sommer B, Sprengel R, Seeburg PH. 1993. Rna Editing of Ampa Receptor Subunit Glur-B - a Base-Paired Intron-Exon Structure Determines Position and Efficiency. *Cell* **75**(7): 1361-1370.

Huntley MA, Lou M, Goldstein LD, Lawrence M, Dijkgraaf GJ, Kaminker JS, Gentleman R. 2016. Complex regulation of ADAR-mediated RNA-editing across tissues. *BMC Genomics* **17**: 61.

Irimia M, Denuc A, Ferran JL, Pernaute B, Puelles L, Roy SW, Garcia-Fernandez J, Marfany G. 2012. Evolutionarily conserved A-to-I editing increases protein stability of the alternative splicing factor Nova1. *RNA Biology* **9**(1): 12-21.

Jain M, Mann TD, Stulic M, Rao SP, Kirsch A, Pullirsch D, Strobl X, Rath C, Reissig L, Moreth K et al. 2018. RNA editing of Filamin A pre-mRNA regulates vascular contraction and diastolic blood pressure. *The EMBO journal*.

Kerpedjiev P, Hammer S, Hofacker IL. 2015. Forna (force-directed RNA): Simple and effective online RNA secondary structure diagrams. *Bioinformatics* **31**(20): 3377-3379.

Liddicoat BJ, Hartner JC, Piskol R, Ramaswami G, Chalk AM, Kingsley PD, Sankaran VG, Wall M, Purton LE, Seeburg PH et al. 2016. Adenosine-to-inosine RNA editing by ADAR1 is essential for normal murine erythropoiesis. *Experimental hematology* **44**(10): 947-963.

Lomeli H, Mosbacher J, Melcher T, Hoger T, Geiger JR, Kuner T, Monyer H, Higuchi M, Bach A, Seeburg PH. 1994. Control of kinetic properties of AMPA receptor channels by nuclear RNA editing. *Science* **266**(5191): 1709-1713.

Lorenz R, Bernhart SH, Honer Zu Siederdissen C, Tafer H, Flamm C, Stadler PF, Hofacker IL. 2011. ViennaRNA Package 2.0. *Algorithms for molecular biology : AMB* **6**: 26.

Maas S, Godfried Sie CP, Stoev I, Dupuis DE, Latona J, Porman AM, Evans B, Rekawek P, Kluempers V, Mutter M et al. 2011. Genome-wide evaluation and discovery of vertebrate A-to-I RNA editing sites. *Biochemical and biophysical research communications* **412**(3): 407-412.

Ohlson J, Pedersen JS, Haussler D, Ohman M. 2007. Editing modifies the GABA(A) receptor subunit alpha 3. *RNA* **13**(5): 698-703.

Srivastava PK, Bagnati M, Delahaye-Duriez A, Ko JH, Rotival M, Langley SR, Shkura K, Mazzuferi M, Danis B, van Eyll J et al. 2017. Genome-wide analysis of differential RNA editing in epilepsy. *Genome research* **27**(3): 440-450.

Yeo J, Goodman RA, Schirle NT, David SS, Beal PA. 2010. RNA editing changes the lesion specificity for the DNA repair enzyme NEIL1. *Proceedings of the National Academy of Sciences of the United States of America* **107**(48): 20715-20719.
